# Supplementary material for: Using digital tools in clinical, health and social care research: a mixed-methods study of UK stakeholders
Source: BMJ Open. 2024 Apr 3;14(4):e076613. doi: 10.1136/bmjopen-2023-076613 (PMC11146398; doi:10.1136/bmjopen-2023-076613)
Supplement: Author's manuscript [file bmjopen-2023-076613.draft_revisions.pdf]

BMJ Open

BMJ Open is committed to open peer review. As part of this commitment we make the peer review history of every article we publish publicly available.

When an article is published we post the peer reviewers' comments and the authors' responses online. We also post the versions of the paper that were used during peer review. These are the versions that the peer review comments apply to.

The versions of the paper that follow are the versions that were submitted during the peer review process. They are not the versions of record or the final published versions. They should not be cited or distributed as the published version of this manuscript.

BMJ Open is an open access journal and the full, final, typeset and author-corrected version of record of the manuscript is available on our site with no access controls, subscription charges or pay-per-view fees (http://bmjopen.bmj.com).

If you have any questions on BMJ Open's open peer review process please email info.bmjopen@bmj.com

BMJ Open

Using Digital Tools in Clinical, Health and Social Care Research: A Qualitative Study of UK Stakeholders

Journal:	*BMJ Open*
Manuscript ID	bmjopen-2023-076613
Article Type:	Original research
Date Submitted by the Author:	12-Jun-2023
Complete List of Authors:	Clohessy, Sophie; University of Warwick, WMG<br>Arvanitis, Theodoros; University of Birmingham<br>Rashid, Umer; University of Warwick, WMG<br>Craddock, Carly; NIHR CRN WM<br>Evans, Mark; NIHR CRN WM<br>Toro, Carla; University of Warwick, Warwick Medical School<br>Elliott, Mark; University of Warwick, WMG
Keywords:	Clinical Trial, QUALITATIVE RESEARCH, Telemedicine < BIOTECHNOLOGY & BIOINFORMATICS

SCHOLARONE™
Manuscripts

I, the Submitting Author has the right to grant and does grant on behalf of all authors of the Work (as defined in the below author licence), an exclusive licence and/or a non-exclusive licence for contributions from authors who are: i) UK Crown employees; ii) where BMJ has agreed a CC-BY licence shall apply, and/or iii) in accordance with the terms applicable for US Federal Government officers or employees acting as part of their official duties; on a worldwide, perpetual, irrevocable, royalty-free basis to BMJ Publishing Group Ltd ("BMJ") its licensees and where the relevant Journal is co-owned by BMJ to the co-owners of the Journal, to publish the Work in this journal and any other BMJ products and to exploit all rights, as set out in our licence.

The Submitting Author accepts and understands that any supply made under these terms is made by BMJ to the Submitting Author unless you are acting as an employee on behalf of your employer or a postgraduate student of an affiliated institution which is paying any applicable article publishing charge ("APC") for Open Access articles. Where the Submitting Author wishes to make the Work available on an Open Access basis (and intends to pay the relevant APC), the terms of reuse of such Open Access shall be governed by a Creative Commons licence – details of these licences and which Creative Commons licence will apply to this Work are set out in our licence referred to above.

Other than as permitted in any relevant BMJ Author's Self Archiving Policies, I confirm this Work has not been accepted for publication elsewhere, is not being considered for publication elsewhere and does not duplicate material already published. I confirm all authors consent to publication of this Work and authorise the granting of this licence.

For peer review only

BMJ Open- Draft 1 SC
26th May 2023

Using Digital Tools in Clinical, Health and Social Care Research: A Qualitative Study of UK Stakeholders

Sophie Clohessy[1], Theodoros N. Arvanitis[2], Umer Rashid[1], Carly Craddock[3,4], Mark Evans[3,4], Carla Toro[5], Mark T. Elliott[1]

[1]Institute of Digital Healthcare, WMG, University of Warwick, Coventry, United Kingdom

[2]School of Engineering, University of Birmingham, Birmingham B15 2TT, UK

[3]The Royal Wolverhampton NHS Trust, Wolverhampton, UK

[4]National Institute for Health Research Clinical Research Network West Midlands, Birmingham, UK

[5]Division of Health Sciences, Mental Health and Wellbeing Group, Warwick Medical School, University of Warwick, Coventry, United Kingdom

Corresponding author's contact details: Mark T. Elliott, email: m.t.elliott@warwick.ac.uk

For peer review only - http://bmjopen.bmj.com/site/about/guidelines.xhtml

BMJ Open- Draft 1 SC
26th May 2023

Abstract

Objective: This mixed-methods study aimed to identify which digital tools are currently used across all stages of clinical research by stakeholders in clinical, health and social care research and investigate their experience using digital tools.

Design: Two online surveys followed by semi-structured interviews were conducted. Interviews were audio recorded, transcribed, and analysed thematically.

Setting, Participants In both online surveys [Researchers and Related Staff (n=41), Research and Development staff (n=25)], participants needed to have worked on clinical, health or social care research studies over the past two years (2020-2022) in an employing organisation based in the West Midlands region of England. Survey participants had the opportunity to participate in an online qualitative interview to explore their experiences of digital tools in greater depth (n=8).

Results: Six themes were identified in the qualitative interviews: '*Definition of a Digital Tool in Clinical Research*'; '*Impact of the COVID-19 Pandemic*'; '*Perceived Benefits/Drawbacks of Digital Tools*'; '*Selection of a Digital Tool*'; '*Barriers and Overcoming Barriers*'; and '*Future Digital Tool Use*'. The context of each theme is discussed, based on the interview results.

Conclusions: Findings demonstrate how digital tools are becoming embedded in clinical research, as well as the breadth of tools used across different research stages. Tools were mostly perceived in a positive light, offering increased efficiency for research. Several considerations were highlighted; concerns about digital exclusion; need for collaboration with digital expertise/clinical staff, research on tool effectiveness and recommendations to aid future tool selection. There is a need for the development of resources to help optimise the selection and use of appropriate digital tools for clinical research staff and participants.

Keywords

Digital Tool, Clinical Research, Clinical Trial, Qualitative, Survey, COVID-19

For peer review only

BMJ Open- Draft 1 SC
26th May 2023

Article Summary

Strengths and Limitations of The Study

- Mixed-method study to explore experiences of utilising digital tools in clinical research, utilising participants from a variety of roles; both Research and Research and Development staff.
- Survey and qualitative questions co-designed with an experienced stakeholder group.
- Qualitative data collection continued until the point in which data saturation was reached; results covered depth as well as a wide number of experiences.
- It is likely that participants were motivated to participate due to an underlying interest in digital tools, consequently some perspectives may not have been included in this study.
- The study was limited to exploring the views of stakeholders employed by organisations based in the West Midlands, United Kingdom.

Introduction

Digital tools are increasingly used across stages of clinical research from recruitment (e.g., social media platforms) to data collection (e.g., online survey platforms) (e.g., Darmawan, Bakker, Brockman, Patten, & Eder, 2020; Gesualdo et al., 2021; Masoli et al., 2021). The COVID-19 pandemic accelerated changes to clinical research methodology, consequently studies were predominantly conducted online and/or remotely (e.g., Chen, Chen, & Chen, 2021; Loucks et al., 2021; Masoli et al., 2021). A report by the UK government [see *'The Future of Clinical Research Delivery: 2022 to 2025 implementation plan*'] sets out a future vision of research delivery which includes data enabled trials (i.e., using electronic health records) and digital tools (Department of Health & Social Care, 2022). Given that digital tools are now commonplace and set to be firmly embedded within clinical research, there is a need to explore people's experiences using digital tools; what has worked well and not so

For peer review only - http://bmjopen.bmj.com/site/about/guidelines.xhtml

well. A recent mixed method study by Blatch-Jones et al. (2020) explored which digital tools were used within recruitment and retention in clinical research in the UK. However, it is evident there is a need to explore experiences of digital tools across the entire breadth of research stages (e.g., informed consent, set up, intervention delivery, management of research studies) as well as investigating the impact of the COVID-19 pandemic on the switch to digital tools for facilitating clinical research. Indeed, a particular focus of this study was on the use and experience of digital tools in the context of the rapid switch to digital methods during the COVID-19 pandemic in the UK between 2020 and 2022.

The aim of this study was two-fold. First, to explore examples of digital tools used by stakeholders across the local geographical region in clinical, health and social care research. Second, to understand how effective digital tools have been for all aspects of clinical research (e.g., set up, management, recruitment, retention, data collection, intervention delivery).

Methods

This study comprised of two main components: *Phase 1:* Online surveys to understand the overall picture of digital tool adoption across the region, with two key stakeholder groups: 1) Researchers and Related Staff 2) Research and Development [R&D] Staff (see Table 1, for the definition of these groups). *Phase 2:* Online survey participants were given the opportunity to participate in qualitative interviews to build upon survey answers, exploring their experiences with digital tools in further depth.

Phase 1: Online surveys

Participants

Two surveys were aimed at the following participant groups, with eligibility criteria shown in Table 1.

Table 1: Eligibility criteria by survey type

Survey Type	Eligibility Criteria
Researchers and Related Staff	People working on clinical research projects over the past two years (2020-2022), either in a research capacity or operational support; aged 18 years or older; employing organisation based in West Midlands region of the UK.

For peer review only - http://bmjopen.bmj.com/site/about/guidelines.xhtml

Research & Development (R&D) Staff	People working within a hospital's Research & Development (or similar) department, with a role to support or coordinate clinical research taking place within, or in partnership with the Hospital over the past two years (2020-2022); 18 years or older; hospital must be a research active NHS Trust in the West Midlands region of the UK.

Recruitment

Participants were recruited primarily using a database of clinical, health and social care research studies held by the National Institute of Health Research Clinical Research Network[1] - NIHR CRN West Midlands. For the Researchers and Related Staff survey, clinical, health or social care projects that had been completed or still ongoing within the past three years[2] were identified by staff within the NIHR CRN West Midlands team. The email invitation and survey link were then distributed to the lead researcher or main contact for the selected research studies. A similar process was followed for the R&D survey, R&D managers were contacted at all West Midlands NHS partner organisations. To further widen the opportunity to participate, a short study description and survey links were advertised on Twitter via the NIHR CRN West Midlands account. After a period of 2 weeks, a follow-up reminder email was sent to all contacts via the same mechanism as the initial survey invitation. Both surveys were live for one month in total. After the first data collection period (27th June - 26th July 2022), participant numbers were lower than expected; therefore, a second phase of recruitment took place (14th October - 15th November 2022). An email prompt was sent to all contacts via the same mechanism as the initial survey invitation.

Procedure

[1] NIHR, a virtual organisation, is the research arm of the National Health Service. The NIHR Clinical Research Network (CRN) is made up of 15 local networks across England which helps patients, the public and health and care organisations to participate in high-quality research (Sydes et al., 2021).
[2] Although, we were primarily interested in including studies which had used digital tools between the years 2020-22 (since beginning of COVID-19), studies were included from the past three years (2019-2022) to ensure a suitable number of studies were included in the survey. Most research was paused to deliver urgent public health research during the pandemic, and then possibly restarted during the two-year window.

For peer review only

For peer review only - http://bmjopen.bmj.com/site/about/guidelines.xhtml

Participants accessed surveys via the Qualtrics platform (https://www.qualtrics.com/uk/). Before taking part, all participants read an information sheet and ticked a consent form to agree to their participation. Before answering any questions, participants were offered a definition of a digital tool and examples of digital tools (See Supplementary Materials, Table S1). The definition varied between the two surveys to account for tools used in a variety of contexts. Survey participants were invited to participate in a qualitative interview study to explore their thoughts towards digital tools in further detail. After survey completion, participants were presented with a debriefing sheet which repeated the objectives of the study and information about how the survey data would be stored.

i. Researchers and Related Staff Survey

Participants were re-directed to the end of the survey if they did not meet the eligibility criteria (see Table 1) determined via initial screening questions. A further screening question determined if participants had used digital tools in their research studies. Participants who answered 'yes' to using digital tools in their clinical, health or social care research were then directed to questions which focused on participants experience of what they perceived to be most effective and least effective digital tools used in their role (blocks presented in a randomised order, questions were the same in each block) (See Supplementary Materials, Table S2 for question themes).

ii. R&D Staff Survey

Participants followed the same procedure as above, except eligible participants answered three blocks of questions about their perceived most effective digital tool used in 1) Set up 2) Recruitment and 3) Management of Clinical Research Studies (blocks were presented in a randomised order, questions were the same in each block). If participants did not have a digital tool example for set up, recruitment or management they skipped this block of questions and were re-directed to the next part of the survey (See Supplementary Materials, Table S2 for question themes).

Data Analysis

For peer review only - http://bmjopen.bmj.com/site/about/guidelines.xhtml

A mixed approach to analysis was undertaken for both surveys. Multiple choice and scale answers were analysed using descriptive statistics; free text answers were analysed thematically. Results from both surveys were analysed, discussed, and verified with the project team.

Phase 2: Qualitative Interviews

Recruitment

During the online survey, participants were invited to take part in the qualitative interviews and expressed their interest by providing their name, job role and email address.

Procedure

All interested participants were sent an electronic information and consent sheet via email and a mutually convenient time and date was arranged via Doodle poll (https://doodle.com/free-poll). Participants returned consent via email; this was also confirmed verbally at the beginning of each interview. Semi-structured interviews were undertaken by the Research Fellow (Author, SC) via Microsoft Teams, lasting between 45-60 minutes (conducted between 20th October- 9th November 2022). The interview topic guides (See Supplementary Materials, Table S3) were informed by the online survey; participants were informed that their survey answers would be discussed in greater detail during the qualitative interviews (referenced on the consent form). All interviews were recorded and transcribed using the built in functionality in Microsoft Teams. Data collection was stopped at the point of data saturation, this was defined as the point of which no new themes were observed.

Data Analysis

Transcripts were anonymised and cross-checked for accuracy by the first author (SC) and Research Assistant (UR) against the recordings. After each interview, key themes were summarised to inform the coding framework. Interview transcripts were then uploaded into NVivo qualitative analysis software (v12, QSR International). A Thematic Analysis was applied, using an inductive approach, meaning that codes were derived from the data. To reduce bias, a second reviewer (UR) independently coded 25% of all interviews using the same method as SC and developed a separate coding framework. To enhance validity, SC and UR discussed the two coding frameworks to produce one final coding

For peer review only - http://bmjopen.bmj.com/site/about/guidelines.xhtml

For peer review only

framework. Themes were identified, reviewed, and defined iteratively. Themes and subthemes were then presented and demonstrated by representative quotes.

Results

Phase 1: Online Survey Results

i. Research and Related Staff Survey Results

A total of 52 people completed the survey, of which eight participants answered that they delegated tool use to colleagues, and therefore were re-directed to the end of the survey. Two participants answered 'no' to having used digital tools; one participant provided reasoning for not using digital tools which was 'stick to what worked in the past' (selected from drop-down menu of answers). If participants only had experience of one digital tool, they were asked questions about this specific example. One participant answered questions about one digital example, however, did not provide the name of the digital tool so therefore this participant was removed from analysis.

A total of 41 participants partially or fully completed the survey (see Supplementary Materials Table S4 for participant characteristics). As an introductory question, participants were asked to list all of the digital tools they have used over the past two years, 11/41 people completed the survey up until this point. Thirty participants continued and provided one digital tool example which they deemed to be 1) the least effective tool and/or 2) the most effective tool, with 19/30 participants answering a number of questions about these tools and completing the survey. Table 2 provides an overview of the most effective and least effective tools by research stage.

Table 2: Overview of most effective and least effective digital tools examples by research stage

Research Stage	Most Effective Examples	Least Effective Examples
Data collection	Castor EDC<br>Electronic completion of questionnaires<br>Electronic database to collect participant data from participating hospitals.<br>MS Teams<br>Online data capture tool<br>Patient Identification Centre (PICS)<br>REDCap tool to upload patient data<br>Teams/Zoom for participant interviews<br>Text messaging	Trial Database<br>Randomisation<br>Database for collected intervention information

For peer review only - http://bmjopen.bmj.com/site/about/guidelines.xhtml

BMJ Open- Draft 1 SC
26th May 2023

Intervention delivery	Smart survey<br>Trial intervention delivered to participants via online web portal	Digital intervention as it requires hardware to be used by the patient/research team
Other	MS Teams	MS Teams
Outcome measures	In house databases	n/a
Participant recruitment	QR code for accessing online survey<br>Social media to recruit participants<br>REDCap e-consent system enables GP practices to remotely consent patients to take part in the trial<br>Twitter page for trial<br>Using an online platform to host consent/demographic forms	Text messaging to invite participants to take part in a survey<br>Facebook and Twitter<br>Asking GP practices to use their SMS systems for inviting patients to questionnaire studies.<br>Health Survey<br>Email - to participants<br>SMS [Text Message]<br>Social media for recruitment<br>Advertising the study's recruitment advert on reputable charities social media accounts<br>Clinical Practice Research Datalink Interventional Research Services Platform system<br>Reports set up to highlight which participants have dropped out in between expressing interest and being randomised.
Research study set up	REDCap<br>WhatsApp<br>MS Teams	Teams<br>Online conferencing tools (dependent on the good connection of all participants)<br>Investigator site file templates distributed via online file sharing service
Quality assurance	[none listed]	Datix
Stage not provided	Castor EDC for sending out surveys to be completed online<br>Remote research meetings with site collaborators	[none listed]

1

For peer review only - http://bmjopen.bmj.com/site/about/guidelines.xhtml

Most tools referenced were from ongoing clinical trials, with the largest number of digital tools referenced from data collection (most effective digital tools) and participant recruitment (least effective). Most tools were widely available, as opposed to a bespoke tool. Notably, some tools (e.g., social media) were listed as both Most Effective and Least Effective tools, highlighting the variation in experiences when using these platforms. Table S5 in Supplementary Information provides a breakdown of participants' experiences of using the most and least effective tools they listed, including training requirements and knowledge of costs. Participants selected a range of 1-5 goals for using digital tools, of which the most popular research goal for using digital tool was "Reduce time/Increase efficiency". The most common users of digital tools were research participants (most effective digital tools) and research delivery (least effective digital tools).

In context of most effective tools, on a scale of 0 - 10 (0 no more effective than other tools to 10 much more effective than other tools) participants gave a mean rating of 8.1±1.3 (see Table S6, supplementary materials). In contrast, the least effective tools, were rated on a scale of 0 - 10 (0 = no less effective than other tools, 10 = much less effective than other tools) with a mean rating of 4.3±2.0. see Table S7, supplementary materials).

For peer review only - http://bmjopen.bmj.com/site/about/guidelines.xhtml

For peer review only

BMJ Open- Draft 1 SC
26th May 2023

ii. Research and Development Survey Results

A total of 25 people completed the survey (see Supplementary Materials Table S8 for participant characteristics) of which three participants answered ‘no’ to having used digital tools; three participants provided reasoning for not using digital tools (selected from a drop-down menu of answers). 22 participants partially or fully completed the survey.

The largest number of most effective tool examples, provided by the R&D survey respondents were for management of clinical studies (N=11). However, the tool referenced in this, and the categories varied substantially, i.e., in most cases, each tool was only mentioned by one participant (see Table 3).Table 4 provides a breakdown of participants’ experiences of using the most and least effective tools they listed, including training requirements and knowledge of costs.

Table 3: Most effective digital tools examples by research stage used by employees working in R&D

Stage of Research	Most Effective Digital Tool Example	N
Set Up	Excel Trackers	1
	informatics	1
	Power BI	1
	SharePoint	1
Recruitment	Social Media platforms	4
	PICS	1
	Contact for Research Database	1
Management	Electronic signing of contracts	1
	Power BI	1
	REDCap	1
	TriNetX	1
	DocuSign	1
	Audit	1
	Study Tracker	1
	MS Teams (data collection)	1
	Online Case Report Form	1
	Patient Trackers	2

For peer review only - http://bmjopen.bmj.com/site/about/guidelines.xhtml

Table 4: R&D staff summary of digital tool use

Question Type	Question Responses	Set Up N (%)	Recruitment N (%)	Management N (%)
Total number of most effective examples	n/a	4	6	11
How did they hear about digital tool	Developed in house	1 (25%)	3 (50%)	4 (36.4%)
	Other	1 (25%)	2 (33.3%)	2 (18.2%)
	Recommended by others	2 (50%)	n/a	2 (18.2%)
	Saw advertised online/social media	n/a	1 (16.7%)	n/a
	Approached by digital tool provider	n/a	n/a	3 (27.2%)
	Total responses	4	6	11
Training required for digital tool	Yes	2 (50%)	2 (33.3%)	7 (63.4%)
	No	2 (50%)	4 (66.6%)	4 (36.6%)
	Total responses	4	6	11
If yes to training, training length	< 1 day	1 (50%)	1 (50%)	4 (57.1%)
	1-5 days	n/a	1 (50%)	n/a
	> 5 days	1 (50%)	n/a	3 (42.9%)
	Total responses	2	2	7
Widely used or bespoke digital tool	Widely used	4 (100%)	5 (83.3%)	8 (72.7%)
	Novel/Bespoke to organisation	n/a	1 (16.7%)	2 (18.2%)
	Novel/bespoke to project team	n/a	n/a	1 (9.1%)
	Total responses	4	6	11
Costs associated with acquiring digital tool	I don't know the answer	1 (25%)	1 (25%)	2 (25%)
	No fee (e.g., open source)	1 (25%)	2 (50%)	2 (25%)
	Periodic recurring fee	1 (25%)	n/a	2 (25%)
	Other	1 (25%)	1 (25%)	2 (25%)
	Total responses	4	4	8

For peer review only

BMJ Open- Draft 1 SC
26th May 2023

Cost effectiveness (1 highly ineffective to 7 highly effective)	3	1 (25%)	n/a	n/a
	4	1 (25%)	n/a	n/a
	5	1 (25%)	4 (100%)	n/a
	6	n/a	n/a	4 (50%)
	7	1 (25%)	n/a	4 (50%)
Total responses		4	4	8

For peer review only

For peer review only - http://bmjopen.bmj.com/site/about/guidelines.xhtml

Phase 2 Results: Qualitative Interviews

A total of eight participants took part in the interviews; five participants had completed the survey for R&D employees, three participants had completed the Researchers and Related Staff survey. Participants were employed by a variety of organisations in the West Midlands area. Six main themes were identified across the transcripts, these are summarised in Table 5 along with selected quotes relating to each theme, discussed in detail below. Two case studies of most effective tools have been summarised in Table S9, supplementary materials; Case Study 1 (Management) and Case Study 2 (Recruitment).

For peer review only

Table 5: Summary of identified themes and representative quotes

Theme	Sub Theme	Example Statements
1. Definition of Digital Tools in Clinical Research		*[1.1] "Anything that you can use online basically. So, it can be a handset, it can be a tablet or a website…it's anything that can connect to the Internet that can ease the process of undertaking research."*<br>*[1.2]"It helps make it easier to collect data…easier to track where research is […] And then thinking about the patient facing role of digital research…giving devices to patients or using patients own devices to actually report outcome measures directly to research teams…using technology, whether it's Internet based technology or sort of phone, tablet, laptop-based technology to hopefully improve research even like an e site file system or a trial master file or trial master file system or just using teams and SharePoint, this is a digital solution."*
2. Impacts of the COVID-19 Pandemic		*[2.1]"We wanted to continue the research where we could do so…we had to move it online. And people just embraced that. We had our IT team. We had enough resources to get everything onto online platforms in order to collect the data on an online questionnaire as opposed to a paper questionnaire."*<br>*[2.2]"We proved that the digital way is working, so that's massive. It definitely gave us the push needed cause we had no choice. People had to start using digital tools and actually once we got through it (Covid) […] you go actually, we're not gonna go back to that way before because this way works really well…and it gave us a lot more buy in, particularly with some nurses who may be resistant to change."*
3. Perceived Benefits/Drawbacks	Perceived Benefits	*[3.1] "For the patients that are happy and IT savvy and things it saves a trip to the post box or whatever to fill in the paper questionnaire if people are happy to do it, especially because it's like transferable, you can answer it on your whether it be a mobile phone or a tablet or a PC."*<br>*[3.2]"We potentially can reach patient populations that wouldn't normally take part in research, those that potentially have a lower mobility, aren't able to travel as much could actually take part in the trial."*<br>*[3.3]"You can ask people not only to talk but also to use the chat, which I think helps some people who are bit kind of less able to interact in a group."*<br>*[3.4]"It's easier on an online tool to have a number of different languages …if you're printing off something that's a hell of a lot of paper…and you're sort of sometimes guessing what language people are wanting things in. If there's a drop-down box that could all of a sudden change it to French or you know Arabic, it's a much easier thing to be able to do."*<br>*[3.5]"WhatsApp has been used to keep contact with researchers between meetings, which I felt was quite effective…so it helps to have a WhatsApp group and say what are you doing? Can I help? It helps between meetings to keep things going."*
	Drawbacks	*[3.6]"In my experience online is good to get a lot of work done rapidly but sometimes you can't read face to face interactions. You can create maybe bonds that are bit beyond the business like you can have a coffee together so there's a little bit of a difference there."*<br>*[3.7]"Some participants might struggle with connection and so on. So, there's a number of technical issues that [could occur] but again that's very similar to a logistical issue that you would have in a face-to-face workshop."*<br>*[3.8]"The further negatives are probably people having to use a new system if they don't use it that often then potentially forgetting how to use it. But obviously we've tried to … reduce that by offering videos, offering…training sessions."*<br>*[3.9]"I think drawbacks are still engagement and I think this is what we're trying to investigate at the moment… what is engagement like across different participant groups and age ranges and demographics and ethnicities and stuff like that… I'm not sure it is inclusive."*
4. Selection of a Digital Tool and Recommendations for Future Digital Tool Use	Selection	*[4.1]"It was basically chosen because it was free, and that's what the university had".*<br>*[4.2]"Things tend to be sort of by word of mouth I think a lot relies upon the fact that you happen to have a conversation you know when somebody says ohh, hold on a minute you could do it this way or you could use this."*
	Recommendations	*[4.3]"Almost a digital checklist and then it points to what program, even if it doesn't say what the program is, it tells you the type of program you need…depending on where you are with it and what you're doing. Are you the sponsor? Well, if you sponsor a study you'll need a very different [tool] to if you're delivering the study… is it multi-site?."*<br>*[4.4]"I think it would be good to have case studies of people who have used some tools and made their own adaptations [for a] service user or participant group."*<br>*[4.5]"If you don't know what's available you, you don't know what you don't know. There could be a digital solution to the problem you've got but if that's not*

For peer review only - http://bmjopen.bmj.com/site/about/guidelines.xhtml

		your background, if you haven't encountered it, you wouldn't know to search for it.... I think a checklist...suggestions or case studies essentially and almost you know sharing best practice across regions…It could be national."<br>*[4.6]"A community of practice might be helpful because then you can bring people together […] and […] exchange information. So, I guess developing a network of practitioners working [on] research using tools… that might help to improve the way we use them."*
5. Barriers and Overcoming Barriers	Barriers	*[5.1]"It feels like people were happy to make the move because you couldn't [use] paper. But following COVID some people have gone back to paper…especially older people […] they said…that they'd be worried about clicking on a link and whether it was, you know, bonafide or not…whether it is safe to click on the link."*<br>*[5.2]"Digital tools are there for everyone. Some teams grab with them and run with them really quickly. Others are really resistant still and I think it's what you know. And it's not clear cut on age at all, but there definitely is a demographic that can feel more comfortable with it, and others that don't and I think that comes down to experience."*<br>*[5.3]"They are trained as nurses first and foremost[…]their first priority is always gonna be their patients and you know, hear stories about them working super long shifts, they don't take a break for the entire time, when are they gonna have time to sit down and play with Excel."*<br>*[5.4]"If they've got no budget for IT, then they'll need to whip up an Excel spreadsheet."*<br>*[5.5]"We've got ideas as to what would make life easier for us, which ultimately makes life easier for staff and for patients […] we don't have the expertise behind it so we would be expecting other people to do it. So, the barrier really is sometimes the experience of putting your idea into something that will actually work. This cost is always going to be a barrier in the NHS…if we don't invest in something, how are we ever going to move forward… and so if we invest in there, then actually we might be able to start, you know, save on staff time. So, I think the biggest barrier is expertise."*
	Overcoming Barriers	*[5.6]"I did a questionnaire study and used 18 practices…I sent a prenotification text to nine practises and nine practices I just sent the link to the study and there was absolutely no difference in participation rate."*<br><br>*[5.7]"We keep an open mind as to whether people want to show their face or not. Because some people are not comfortable with showing their house, you know it's quite invasive in a way […] if some people want to keep the cameras on, we say we prefer them to keep the camera on. But if they want to keep off and just participate by audio [we're] also quite open to that."*<br><br>*[5.8]"There's a potential that maybe people who aren't technologically aware would struggle to take part in the study […] it isn't an all or nothing because this is a remote study it has to be remote, no you can actually take that remote nature away and just have a patient complete it on a practice computer."*
6. Future Digital Tool Use	N/A	*[6.1]"Maybe in 20 years and everybody will really use them. So digital exclusion might reduce over time I feel."*<br>*[6.2] "Clinical trials seem to be becoming more complex […] I think people are more inclined to look at digital support with studies […] I think historically […] nurses would be expected to pick up these sorts of administrative tasks. I think now we're looking at getting people in who can do it and it's a lot more efficient."*<br>*[6.3]"Researchers here come and say to me…what's your evidence for using it? What's are your response rates for those via paper and those that answered via e-mail or text message? […] I just think that needs to be published a bit more so people can make informed decisions around what they're going to use for their particular sample."*

For peer review only - http://bmjopen.bmj.com/site/about/guidelines.xhtml

Theme 1: Definition of Digital Tools in Clinical Research

Participants perceived a digital tool used within clinical research as a device that can connect to the internet, or a form of online technology. The main reasoning cited for using a digital tool was to increase efficiency within research (Table 5, Quotes 1.1-1.2). Digital tools have been adopted across all stages of clinical research from recruitment to data collection, it was noted that tools may differ depending on the research stage and/or role (e.g., R&D staff may encounter different digital tools compared to research participants).

Theme 2: Impact of the COVID-19 Pandemic

It is evident that the COVID-19 pandemic accelerated the use of digital tools within clinical research. It was noted how changes to research methodology needed to be implemented quickly; moving from paper-based methods to digital methods. Despite changes in working practices, participants perceived attitudes among staff and research participants to be mostly positive towards tools. COVID-19 was perceived as a time to illustrate the benefits of digital tools, helping to alter stakeholder's views who may have been more pessimistic towards tools prior to their increased use due to COVID-19 (Table 5, quotes 2.1-2.2.)

Theme 3: Perceived Benefits and Drawbacks of Digital Tools

3.1. Benefits

Benefits of tools were considered from two perspectives; research participants and staff using digital tools. For research participants, perceived benefits of using tools included convenience and increased opportunities to take part in research. It was also noted that digital tools can account for individual differences of research participants; for example, some research participants may feel more comfortable contributing to a focus group using the chat function on Microsoft Teams rather than

For peer review only

speaking aloud. From the perspective of staff, interview participants considered efficiency (e.g., clinical delivery staff spending less time on admin tasks leading to more time with trial participants), easy connectivity between colleagues (particularly if based in different locations) and ease of offering research materials in different languages as primary benefits of digital tools (see Table S9, Case Study 2 for most effective digital tool for recruitment). (Table 5, quotes 3.1-3.5).

3.2. Drawbacks

Drawbacks of digital tools included technical issues; for example, poor internet connection or researchers may require additional technical support. However, it was noted that face-to-face research studies may also require additional support. Other drawbacks included difficulty reading interactions online. Despite the ease of meeting with colleagues online, especially when based in different locations and organisations, there was some concern that meeting colleagues online does not always enable people to strengthen or develop relationships in the same way as meeting face to face allows. A further drawback raised was engagement with studies that use digital tools, it was noted that levels of engagement are currently unclear from different research participant groups when using various digital tools. Digital literacy was identified as a potential risk to excluding certain people with a lack of familiarity with using digital tools, especially if research studies are conducted solely using digital tools (Table 5, quotes 3.6-3.9).

Theme 4: Selection of a Digital Tool and Recommendations for Future Digital Tool Use

4.1. Selection

Most participants cited having little choice in the digital tool they used, this was mainly due to the fact that most tool examples were widely used in an organisation (e.g., Microsoft Teams) and as a result they did not have the authority to select a tool. Despite this, it was recognised that familiarity with widely used tools was beneficial for ease of use. There was a concern that often staff are not aware

For peer review only

of what the most appropriate tool would be for a research task. Some bespoke tools were utilised; two participants described the process of designing and developing a bespoke digital tool in collaboration with clinical staff (see Table S9, case study 1 for most effective digital tool for management). The most common way staff discovered about digital tools appears to be through word of mouth (Table 5, quotes 4.1-4.2)

4.2. Recommendations for future tool selection

A number of recommendations were proposed to assist staff in future tool selection. There appears to be no standardised process outlining how to choose a tool, therefore a checklist database was proposed in which staff could input their digital tool requirements (e.g., research stage, user type) and a programme could provide appropriate digital tool options. Further recommendations included a central place (e.g., website) to share information about tools, case studies of effective tools (by research stage/stakeholder group) and a local/national network to share digital tool expertise (Table 5, quotes 4.3-4.6)

Theme 5: Barriers and Overcoming Barriers

5.1. Barriers

Several barriers to digital tool use were referenced including attitudes of staff and research participants; one participant explained that older research participants were concerned about the legitimacy of receiving a survey link via email. A commonly cited barrier was the conflict of carrying out a patient centred job (i.e., nurse) whilst finding time to use digital tools. Resources were also cited as a barrier to digital tool use; resources included budget, physical resources (e.g., poor internet connection or limited digital equipment), digital expertise (some organisations had access to specialist digital teams embedded in a Research and Development department) as well as digital literacy (research participants and staff). Available resources varied across organisations and research

For peer review only - http://bmjopen.bmj.com/site/about/guidelines.xhtml

participants; appearing to be both a key barrier as well as a key driver for digital tool use (Table 5, quotes 5.1-5.5).

5.2. Overcoming Barriers

A number of ways to overcome barriers were proposed by interview participants. Some reported providing research participants with a physical location to visit to complete research activities on a computer (if they did not have access to a computer and/or internet connection). Other participants reported using a hybrid approach of both paper and digital tools to carry out the same research tasks. A hybrid approach was undertaken to reduce digital exclusion, catering for research participants who may not have access to digital tools/internet and/or prefer paper-based methods. However, an impact of this approach included a larger workload due to increased data management.

It is evident that individual differences exist with digital tool use, with interview participants described adopting a flexible approach with research participants. For example, some research participants may not feel comfortable using their video camera when using Microsoft Teams. Interviewees explained how research participants and staff attitudes can be a barrier to digital tool use but offering training in multiple modalities can help to increase users' confidence (e.g., video, face-to-face demos). Some interviewees mentioned conducting their own research to evaluate the effectiveness of digital tools. For example, one participant had previously compared two recruitment methods; a text message in advance informing participants they would receive a survey link via email compared to no pre-notification at all. However, this study reported no differences in the number of participants recruited to the study via either method, suggesting that reassurance of the legitimacy of the survey link did not have any impact in terms of increasing engagement (Table 5, quotes 5.6-5.8).

Theme 6: Future Digital Tool Use

For peer review only - http://bmjopen.bmj.com/site/about/guidelines.xhtml

All participants perceived they would be using tools increasingly in the future and that digital exclusion of research participants may reduce over time due to increasing familiarity with digital tools. Participants highlighted the potential for pressure to be placed on nurses to keep up to date with digital tools whilst caring for patients, as well as the need for collaboration between digital experts and clinical staff when developing and utilising tools. Suggestions for research to inform future tool use included investigating which tools are most effective for different research stages and stakeholder/participant groups and research exploring the cost effectiveness of digital versus paper-based tools. Further suggestions included organisations conducting training analysis of all stakeholders using digital tools (Table 5, quotes 6.1-6.3).

Discussion

This mixed-method study aimed to explore which tools are being used across clinical research as well as staff experiences of digital tool use. Our findings indicate that digital tools are now a fundamental part of clinical research; the COVID-19 pandemic accelerated the use of tools with adoption observed across all research stages (e.g., Barakat et al., 2021; Everhart et al., 2021; Fisher et al., 2021; Masoli et al., 2021). Tools were mostly viewed favourably, with increased efficiency cited as a leading benefit for research staff. It was noted that tools can account for individual differences of research participants, (e.g., materials can be easily offered to research participants in different languages and reduce participants need to travel to a physical location), potentially offering research opportunities to a broader sample of participants (e.g., Blatch-Jones et al., 2020; Masoli et al., 2021; Rosa, Marsch, Winstanley, Brunner, & Campbell, 2021; Sydes et al., 2021). Despite this, some drawbacks, and barriers to the use of tools exist. In particular, there were concerns about digital exclusion of research participants who may not have access to digital tools, and/or internet connections, or lack familiarity with using these platforms (e.g., Alsem et al., 2019; Brennan et al., 2022; Carpenter et al., 2021; Masoli et al., 2021). There was also a concern about limited resources

For peer review only

within clinical research; particularly the need for individuals with digital expertise to advise on and develop digital tools.

A previous mixed methods study by Blatch-Jones et al. (2020) explored UK stakeholders experiences of using digital tools for recruitment and retention within clinical trials. The current study extends this research by considering digital tool use in the context of the COVID-19 pandemic as well as across a wider range of research stages (i.e., informed consent, management, data collection), considering not only the benefits, but also the limitations of digital tools. Consistent with the afore mentioned study by Blatch-Jones et al. (2020), our findings add to existing evidence that digital tools provide opportunities to widen reach of participation as well as reflecting previous concerns around digital exclusion for some groups (Blatch-Jones et al., 2020; Masoli et al., 2021). One of the new insights from the present study includes suggestions for overcoming barriers to digital tool use. Recommendations included attempts to reduce digital exclusion by providing training on digital tools in various modalities (e.g., videos, face-to-face) to increase research participants/staff confidence in use and providing a physical location and/or digital tool in order for people to participate in a study. Further insights included issues around digital tool selection, participants proposed the need for a standardised process for selecting the most appropriate tool as well as published case studies of effective tools. Indeed, it has been previously reported that staff are often not sure of tool appropriateness for a particular task, research stage or stakeholder group and therefore a formalised selection process is required (Blatch-Jones et al., 2020; Masoli et al., 2021). The need for collaboration with clinical staff and digital expertise was also highlighted in the context of developing digital tools in the future, case study 1 (Table S9, Supplementary materials) provided an example of a cost-effective bespoke tool.

Concerns exist around the security of data when using digital tools, particularly among older age research participants and invitations to participate via text/email (Frampton, Shepherd, Pickett, Griffiths, & Wyatt, 2020; Rosa et al., 2021). It is important that research participants perceive

For peer review only - http://bmjopen.bmj.com/site/about/guidelines.xhtml

invitations as authentic and legitimate otherwise this may negatively affect study uptake (Blatch-Jones et al., 2020). In the present study, a participant reported conducting research to compare the recruitment rates of a pre-notification text to inform participants they would be shortly receiving a survey link compared with no text. However, no difference was found between the methods on participant uptake. This indicates such an approach may not be effective in improving the credibility of the subsequent message. It is critical that participants can trust recruitment invitations for clinical research, with the opportunity for scam messages being minimised, based on the way invitations are distributed.

This study highlights several considerations for research staff and organisations when utilising digital tools for research. Research projects should aim to be inclusive for people who may not have access to digital tools or the internet (e.g., providing a physical location/digital tool for people to complete the study) (Masoli et al., 2021). Organisations need to consider whether adequate training is provided for both staff and research participants to help address gaps in knowledge, demystify tools and increase confidence (Masoli et al., 2021). It is apparent that one size does not fit all, research staff should be willing to accommodate personal preferences of research participants when using digital tools (e.g., option to keep video camera off on video conferencing platforms) as well as consider offering a hybrid approach (both paper and digital tool options). In addition, staff should capitalise on digital expertise both locally in their organisations and nationally via funders and other relevant organisations.

Strengths of this study include the use of a broad range of stakeholders as participants and the use of mixed methods, enabling exploration of digital tool use from multiple perspectives. However, a limitation of this study is not exploring the views of participants/carers. Given that our findings alongside previous research suggest that participants/carers are a key user of digital tools, future research should seek to understand their experiences. It is evident that future research should develop a number of resources to help researchers choose appropriate digital tools. Our research

For peer review only - http://bmjopen.bmj.com/site/about/guidelines.xhtml

findings alongside previous studies have recognised requirements to publish case studies of effective digital tools (specific to research stage/stakeholders) as well as develop a standardised process of choosing a tool and/or programme for recommending appropriate tools (Sydes et al., 2021). A target for the funders and organisations related to clinical research is to develop local and national networks to share and showcase expertise in digital tools. Further research avenues should include an evaluation of the effectiveness of tools (specific to the research stage) and an economic analysis to better understand the cost effectiveness of digital tools (particularly in relation to non-digital approaches) which would help to bolster the argument for use of digital tools in clinical research.

References

Alsem, M. W., Verhoef, M., Braakman, J., van Meeteren, K. M., Siebes, R. C., Jongmans, M. J., . . . Www, r. s. g. (2019). Parental empowerment in paediatric rehabilitation: Exploring the role of a digital tool to help parents prepare for consultation with a physician. *Child: care, health and development, 45*(5), 623-636.

Barakat, S., Touyz, S., Maloney, D., Russell, J., Hay, P., Cunich, M., . . . Maguire, S. (2021). Supported online cognitive behavioural therapy for bulimia nervosa: a study protocol of a randomised controlled trial. *Journal of eating disorders, 9*(1). doi:10.1186/s40337-021-00482-w

Blatch-Jones, A., Nuttall, J., Bull, A., Worswick, L., Mullee, M., Peveler, R., . . . Griffiths, G. (2020). Using digital tools in the recruitment and retention in randomised controlled trials: Survey of UK Clinical Trial Units and a qualitative study. *TRIALS, 21*(1). doi:10.1186/s13063-020-04234-0

Brennan, C. M., Nadella, S., Zhao, X., Dima, R. J., Jordan-Martin, N., Demestichas, B. R., . . . Janowitz, T. (2022). Oral famotidine versus placebo in non-hospitalised patients with COVID-19: a randomised, double-blind, data-intense, phase 2 clinical trial. *Gut*.

Carpenter, M. J., Gray, K. M., Wahlquist, A. E., Cropsey, K., Saladin, M. E., Froeliger, B., . . . Dahne, J. (2021). A Pilot Randomized Clinical Trial of Remote Varenicline Sampling to Promote Treatment Engagement and Smoking Cessation. *Nicotine & tobacco research : official journal of the Society for Research on Nicotine and Tobacco, 23*(6), 983-991.

Chen, Z. M., Chen, L. R., & Chen, H. F. (2021). The impact of COVID-19 on the clinical trial. *PLOS ONE, 16*(5). doi:10.1371/journal.pone.0251410

Darmawan, I., Bakker, C., Brockman, T. A., Patten, C. A., & Eder, M. (2020). The Role of Social Media in Enhancing Clinical Trial Recruitment: Scoping Review. *J Med Internet Res, 22*(10), e22810. doi:10.2196/22810

Department of Health & Social Care. (2022). *The Future of Clinical Research Delivery: 2022 to 2025 implementation plan*.

Everhart, R. S., Dempster, K. W., Withers, D., Mazzeo, S. E., Corona, R., Thacker, L. R., 2nd, & Schechter, M. S. (2021). Adaptations to the RVA Breathes clinical trial due to the COVID-19 pandemic. *CONTEMPORARY CLINICAL TRIALS COMMUNICATIONS, 24*, 100871.

Fisher, L. B., Tuchman, S., Curreri, A. J., Markgraf, M., Nyer, M. B., Cassano, P., . . . Pedrelli, P. (2021). Transitioning From In-Person to Remote Clinical Research on Depression and Traumatic

For peer review only - http://bmjopen.bmj.com/site/about/guidelines.xhtml

Brain Injury During the COVID-19 Pandemic: Study Modifications and Preliminary Feasibility From a Randomized Controlled Pilot Study. *JMIR formative research, 5*(12), e28734.

Frampton, G. K., Shepherd, J., Pickett, K., Griffiths, G., & Wyatt, J. C. (2020). Digital tools for the recruitment and retention of participants in randomised controlled trials: a systematic map. *TRIALS, 21*(1), 478. doi:10.1186/s13063-020-04358-3

Gesualdo, F., Daverio, M., Palazzani, L., Dimitriou, D., Diez-Domingo, J., Fons-Martinez, J., . . . Tozzi, A. E. (2021). Digital tools in the informed consent process: a systematic review. *BMC medical ethics, 22*(1). doi:10.1186/s12910-021-00585-8

Loucks, T. L., Tyson, C., Dorr, D., Garovic, V. D., Hill, J., McSwain, S. D., . . . Brady, K. T. (2021). Clinical research during the COVID-19 pandemic: The role of virtual visits and digital approaches. *JOURNAL OF CLINICAL AND TRANSLATIONAL SCIENCE, 5*(1), e102.

Masoli, J. A. H., Down, K., Nestor, G., Hudson, S., O'Brien, J. T., Williamson, J. D., . . . Carroll, C. (2021). A report from the NIHR UK working group on remote trial delivery for the COVID-19 pandemic and beyond. *TRIALS, 22*(1). doi:10.1186/s13063-021-05880-8

Rosa, C., Marsch, L. A., Winstanley, E. L., Brunner, M., & Campbell, A. N. C. (2021). Using digital technologies in clinical trials: Current and future applications. *CONTEMPORARY CLINICAL TRIALS, 100*, 106219.

Sydes, M. R., Barbachano, Y., Bowman, L., Denwood, T., Farmer, A., Garfield-Birkbeck, S., . . . Valentine, J. (2021). Realising the full potential of data-enabled trials in the UK: a call for action. *BMJ OPEN, 11*(6), e043906. doi:10.1136/bmjopen-2020-043906

Acknowledgements The authors thank all study respondents taking part in the study. The authors also thank the steering group for their contributions as well as the participants who provided case study examples of effective digital tools.

Contributors CC, TNA, CT and MTE obtained funding and conceptualised the research. ME and SC drafted initial online survey questions and qualitative interview questions; SC, CC, TNA, ME, MTE, CT, reviewed the draft survey/qualitative questions. SC, ME, CC, MEV were directly involved in activities relating to participant recruitment. SC and OR tidied the qualitative interview transcripts and analysed qualitative data. SC and MTE conducted data analysis for online survey results and drafted the manuscript, all authors reviewed the final manuscript.

Funding This study was supported by the National Institute for Health and Care Research, Clinical Research Network West Midlands Improvement, and Innovation Strategic Funding.

Competing interests CC and MEV are employed by the funder (NIHR) of this project. The review is part of a wider project which CC helped to conceptualise. CC and MEV have revised and helped finalise the online survey and interview questions while also providing guidance about digital tools used in clinical research. CC and MEV were directly involved in activities relating to participant recruitment.

Patient and public involvement No patients and/or public were involved in the design or conduct or reporting or dissemination plans of this research.

Patient consent for publication Not required.

Ethics Approval This study involves human participants and was approved by the University of Warwick's Biomedical and Scientific Research Ethics Sub-Committee (111/20-21). Participants gave informed consent to participate in the study before taking part.

For peer review only - http://bmjopen.bmj.com/site/about/guidelines.xhtml

Supplementary Material

Table S1: Definition of digital tool by survey type

Research Staff	Research and Development
In this study a **digital tool** is defined as an alternative to paper-based methods that is IT based or an online platform that aids any aspect of the research study (stages of clinical research include but are not limited to; set up of research studies, participant recruitment, consent, participant retention, intervention delivery, data collection, data analysis, monitoring, patient reported outcomes).<br>Examples of digital tools might include but are not limited to:<br>• Electronic databases for participant screening<br>• Social media platforms for participant recruitment<br>• Communication within your research team (e.g., reporting adverse events via text message or email)<br>• Electronic consent<br>• Text messaging for participant retention<br>• Digital platforms for collecting survey data	In this study a **digital tool** is defined as an alternative to paper-based methods that is IT based or an online platform that aids any aspect of the research study set up, recruitment of participants and management.<br>Examples of digital tools might include but are not limited to:<br>• Electronic screening for participants at site level<br>• Remote monitoring via smart phone applications<br>• Local social media routes<br>• R&D department's website<br>• Text alerts

For peer review only

Table S2: Summary of Question area by survey type (Research Staff, R&D)

Survey Type	Question Themes
**Research Staff**	**Most effective digital tool**
	· Description of tool
	· Type of research digital tool used within (e.g., clinical trial)
	· Stage of research project tool related to (e.g., recruitment)
	· Project status
	· Widely used/bespoke tool
	· Costs associated with acquiring tool
	· Training required to use tool
	· End user of tool (drop down menu of pre-specified answers)
	· Effectiveness of the digital tool relative to other digital tools (0 = not effective, 10 = very effective)
	· How essential was digital tool to successfully implement the research (relative to alternative non-digital tools) (1 Highly non-essential to 5 Highly essential)
	· Effectiveness of integration of digital tool with other health care or research systems (1 Not applicable to 5 highly effective)
	· Level of technical literacy required to use/access tool (1 Very difficult to use/access to 4 Very easy to use/access)
	· Main goal/s of tool (drop down menu of pre-specified answers)
	Least effective digital tool
	· Same as above except for Ineffectiveness of the digital tool relative to other digital tools 0 = no less effective than other tools, 10 = much less effective than other tools.
**R&D**	**Most effective digital tool example for set up**
	· Description of tool
	· How heard about digital tool
	· Training (if yes number of days)
	· Widely used/bespoke novel tool
	· Cost information
	· Perceived cost effectiveness of tool
	Most effective digital tool example for recruitment
	· Same as above
	Most effective digital tool example for management

For peer review only

	· Same as above

Table S3: Summary of interview question areas by participant type

Interview Type	Question Themes
Researchers and Related Staff	Definition of digital tools<br>How did COVID-19 affect role/use of digital tools<br>Case study about most effective tool<br>Case study about least effective tool<br>Any instances where tools could be used<br>Any barriers to digital tools<br>What would help researchers when deciding what tool to use/where to find this information
R&D Staff	Definition of digital tools<br>How did COVID-19 affect role/use of digital tools<br>Case study most effective tool for set up<br>Case study most effective tool for recruitment<br>Case study most effective tool for management<br>Any instances where tools could be used<br>Any barriers to digital tools<br>What would help researchers when deciding what tool to use/where to find this information

Table S4: Research staff and related survey participant characteristics

Number of Participants (N=41)	N (%)
County	
County of the West Midlands	26 (63.4)
Warwickshire	9 (22)
Staffordshire	4 (9.8)
Multiple counties in the CRN West Midlands	2 (4.9)

For peer review only

For peer review only - http://bmjopen.bmj.com/site/about/guidelines.xhtml

Employing organisation	
University	14 (34.1)
Clinical Trials Unit	13 (31.7)
NHS Trust	11 (26.8)
Other (please provide further details below)	3 (7.3)
Job role	
Chief Investigator	8 (19.5)
Other	8 (19.5)
Trial/Study Coordinator	8 (19.5)
Trial/Study Manager	8 (19.5)
Research Manager	3 (7.3)
Principal Investigator	2 (4.9)
Research Nurse	2 (4.9)
Trial/Study Data Manager	2 (4.9)

1

2 **Table S5** Most effective and least effective digital tools results

Question Type	Answer Options	Most Effective N(%)	Least Effective N (%)
Type of research project	Clinical Trial	8 (38.1)	9 (42.9)
	Clinical Research	6 (28.6)	6 (28.6)
	Health Research	5 (23.8)	4 (19)
	Other	1 (4.8)	1 (4.8)
	Social Care	1 (4.8)	1 (4.8)
	Total number of responses	21	21
Stage of research	Research study set up	2 (9.5)	4 (19)
	Participant recruitment	4 (19)	8 (38.1)
	Data collection	11 (52.4)	6 (28.6)
	Intervention delivery	2 (9.5)	1 (4.8)
	Quality Assurance	n/a	1 (4.8)
	Outcome measures	1 (4.8)	n/a

For peer review only

	Other	1 (4.8)	1 (4.8)
	Total number of responses	21	21
Project status	Completed	4 (19)	4 (19)
	Ongoing	17 (81)	17 (81)
	Total number of responses	21	21
Widely used vs Bespoke digital tool	Novel/bespoke to my organisation	4 (19)	3 (14.3)
	Novel/bespoke to my project team	5 (23.8)	1 (4.8)
	Used widely	12 (57.1)	17 (81)
	Total number of responses	21	21
Costs associated with acquiring digital tool	A one-off up-front fee	1 (4.8)	1 (4.8)
	A one-off up-front fee, periodic recurring fee	2 (9.5)	1 (4.8)
	I'm not sure	4 (19)	8 (38.1)
	No fee (e.g., open source)	8 (38.1)	7 (33.3)
	Other	3 (14.3)	3 (14.3)
	Periodic recurring fee	3 (14.3)	1 (4.8)
	Total number of responses	21	21
Training required in order to use tool	No	11 (52.4)	12 (57.1)
	Yes	10 (47.6)	9 (42.9)
	Total number of responses	21	21
How essential was this tool to successfully implement the research	Somewhat non-essential (2)	1 (4.8)	4 (19)
	Somewhat essential (3)	8 (38.1)	9 (42.9)
	Highly essential (4)	12 (57.1)	8 (38.1)
	Total number of responses	21	21
Integration with other healthcare or research systems	Not applicable (1)	3 (14.3)	2 (9.5)
	Somewhat ineffective (3)	3 (14.3)	6 (28.6)
	Somewhat effective (4)	8 (38.1)	10 (47.6)
	Highly effective (5)	7 (33.3)	3 (14.3)
	Total number of responses	21	21

For peer review only

Level of technical literacy required	Somewhat difficult to use/access (2)	1 (4.8)	7 (33.3)
	Somewhat easy to use/access (3)	10 (47.6)	10 (47.6)
	Very easy to use/access (4)	10 (47.6)	4 (19)
	Total number of responses	21	21

Table S6: Rating of effectiveness of most effective tool (0 no more effective to 10 much more effective)

Answer Options		Rating of most effective tool compared to other tools
	0	n/a
	1	n/a
	2	n/a
	3	n/a
	4	n/a
	5	1 (4.8)
	6	1 (4.8)
	7	4 (19)
	8	7 (33.3)
	9	5 (23.8)
	10	3 (14.3)
Total number of responses		21

Table S7: Rating of ineffectiveness of least effective tool (0 = no less effective than other tools, 10 = much less effective than other tool)

For peer review only

Answer Options	Rating of least effective tool compared to other tools
0	1 (5%)
1	1 (5%)
2	2 (10%)
3	2 (10%)
4	3 (15%)
5	7 (35%)
6	1 (5%)
7	2 (10%)
8	1 (5%)
9	n/a
10	n/a
Total number of responses	20

For peer review only

Table S8: R&D Survey Participant Characteristics

Number of Participants (N=25)	N(%)
Experience Using Digital Tools	
Participants who used digital tools	22(88)
Participants who have not used digital tools	3(12)
County	
County of the West Midlands	10(40)
Warwickshire	5(20)
Multiple counties in CRN West Midlands	3(12)

Staffordshire	3(12)
Worcestershire	2(8)
Herefordshire	1(4)
Shropshire	1(4)
Employing Organisation	
NHS	25(100%)
Job Role	
R&D Manager/Head of R&D	13(52)
Lead Research Nurse	3(12)
Other (please provide further details below)	3(12)
R&D Facilitator (governance, study set-up)	3(12)
Data Manager	1(4)
Research Delivery staff	1(4)

1

For peer review only

BMJ Open- Draft 1 (SC)
May 2023

Table S9: Most effective tool case studies for management and recruitment

**Case study 1: Digital Tool for Management**	**Example Statements**
Two participants referred to a bespoke management tool as their most effective digital tool. The tool was designed and developed by R&D staff supported by a digital team embedded in an R&D department in collaboration with clinical research staff (primarily nurses). Built in excel, the tool is used to manage patients through a study protocol. Below, one participant describes the tool.	*"It meets multiple needs, so we build them individually off the back of each protocol…so when you put the patients recruited date it then predicts all their follow up visits for you in keeping with the protocol. It then has a traffic light system which would tell you when they're due to come up so the teams can see it and have oversight quite quickly of all the participants where they are, what happens at each individual stage. You then hover your mouse over visit 2 and a little info box will come up and tell you a brief synopsis of what needs to be done at that visit. So going quickly, either give you what needs to be done, you can click on it, and it takes you through to the worksheets that we create off the back of it."*
One participant explained the primary reason for developing the tool was to standardise study management.	*"It came around a sort of a want to offer standardised high-quality research and oversight... I've worked in lots of different research areas, and they've always got slightly different way of working. Some of the nurses and the clinical teams have sort of had to go at building their own Excel spreadsheets. Some of the sponsors and the studies provided them for us and then some used paper diaries. So, but when you look at it, none of them were quite really fulfilling everyone's needs, there were sort of doing a bit here and a bit there and I wanted to standardize it across the whole of R&D because it wasn't quite equitable for our patients and our staff. Some were getting access to. So that's why we standardized and went we're gonna build these and offer them out across the board."*
Prior to development of this bespoke management tool, it appears that studies were managed via paper means and/or nurses had attempted to build their own databases to manage studies.	*"Nurses that trying to build databases, you know, it's not our skill set, but some can do sort of just a small amount with them and then others were very much using paper which is not the way to do it."*
Both participants deemed the bespoke management tool to be effective for a number of different reasons including helping staff keep patients to the protocol time frames. One participant explained that the tool can be tweaked to suit needs of the users (i.e., nurses) and new features can be added. They also explained that it has many benefits due to being developed in excel, including tool template can be sent to teams located at different sites and used without the R&D staff visiting that particular site as well as being user friendly.	*"In terms of what it gains and what it offers for clinical deliveries…if you use [tool name] your patient should stay within [the] protocol visit window…and you can plan a lot easier, and again should someone go off…someone can come in and pick it up quite easily and you can ensure the patient is safe and kept up to date."*<br><br>*"One of the benefits of it is we've built it in Excel. It doesn't look like excel when you use it. Everyone's got excel within the trust, and there's no additional cost apart from the hours to build it, we weren't having to purchase another system. So that was why we chose that setup to build it. And then we're working with other sites and helping them have access. We can send them a template, we don't need to go there…they upload the template, then they can use it themselves. So that's why we built it that way just for that reason."*<br>*"I think we're on version seven or eight now, so they're really user friendly and they're really locked down so you can't break them. I've had some nurses do some really weird things and they've broken [it] but generally as times gone by, we've ironed out all the things. Before if a nurse dragged a formula*

For peer review only

	away and deletes it, it would affect everything else…but in the department we've [become] more savvy at locking things down. Generally, …you should be able to click and use it."
A further benefit highlighted by participants is the fact that the tool was developed in collaboration with clinical research staff and has incorporated their ideas into the tool such as automatically generating reports opposed to looking through paper documents.	*"It's made by the people delivering research…I think together we've developed the service over the years and the tracker itself and that's what makes it better than an out-of-the-box bit of kit from it or third-party developer…one of the things we built in which was one of the ideas from a nurse, she had nearly a 1000 patients on a study but she just wanted to see who she needed to see this month, and rather than scrolling down the 1000 patients with a pen seeing "Who do I need to see?" She clicks the month button, and it will automatically generate your report in PDF and just say these are your patients for the month…I think that's really where the track of kind of stands out. It's just that bit of being able to organize and manage yourself and your team a lot more effectively and actually you don't want to spend time trawling through bits of paper, paper calendars and diaries."*
**Case Study 2: Digital Tool for Recruitment**	**Example Statements [Participant Number]**
One participant described digital methods used in a clinical trial to recruit participants who meet the inclusion criteria of a study. Using data from the CPRD, researchers are able to know how many patients within each GP surgery meet an inclusion criteria for a study. Advantages of this include reduction in time and money spent on recruitment, as well as increases the likelihood of recruiting relevant participants. It is also beneficial for the patient, as the GP surgery may have relevant information on file that research studies can use (e.g., blood pressure). This is in turn also reduces the workload for the research team.	*"Practices sign up to CPRD [Clinical Practice Research Datalink] and they've got around 20% of practices across England. So CPRD are able to extract their coded medical data. So, any information that when you go to the GP and they say you've got diabetes, they put what they call a medical code in your patient records. So, you've got diabetes… CPRD able to extract that medical code...it's just that coded that this patient has diabetes, their blood pressure was this, they're on these drugs. They're able to extract that from practice records. So CPRD and [employing university] have spotted that actually there's a potential for using this in an interventional trial. So, it means there is an ability to extract that medical info, we can first of all pre-screen remotely the entire of CPRD's population, so over 13 million records we're able to pre-screen against our trial inclusion and exclusion criteria. So, we're then able to actually instead of the old-fashioned research way, which is you ask practices that do they want to take part in research? They say yes. Sounds interesting. You set them up, you spend time, money, setting them up, and actually they've got one patient…they haven't got the patients you need. Whereas with this ability of CPRD's, we're able to know that practice has this amount of patients…we're going to those practices that are large and potentially successful in actually taking part in research so that means obviously it reduces the time that we spend, the money that we spend and hopefully increases the success of the trial…and also obviously reduces practice workload... every so often a patient would go into the practice, have their blood pressure taken anyway for us then to ask that patient to come in for an extra research visit when actually we can just pull that data from the medical record, it seems counterintuitive, so making it easier one for practices they don't have to arrange for patients to come in and two for patients, it means they can absolutely take part in this trial without actually having to go into their GP practice."*

For peer review only - http://bmjopen.bmj.com/site/about/guidelines.xhtml

BMJ Open- Draft 1 (SC)
May 2023

The participant explained that they also used e-consent in the trial when recruiting participant. Consent forms were sent to patients from the GP practises via text message or email. The participant explains the process below.	*"In response to COVID and the reduction in face-to-face GP visits we obviously needed to find a work around practices to be able to recruit patients to the trial. So, we actually developed a fully remote consent system using REDCap…we used it to send e-consent forms out to patients. So, the GP practice puts in some contact details of the patient and that then automatically sends a text message or e-mail to the patient containing a link to their consent form in this sort of electronic survey form. Patients then click on that link in that text message or e-mail it brings up their e-consent form. It provides them with the patient information sheet that they need to complete. Then they then go down and click yes, yes or no to each of the consent criteria. They then fill in their details. They're able to electronically sign that with that signature being saved so it matches that, that sort of the NHS criteria for e-consent system we then collect further information, so their e-mail and telephone number.*
The participant explains below the benefits of participants, GP and research team all receiving a copy of the e-consent form	*"and then once they've completed that it automatically notifies the GP practice that they've completed…the GP practice are then able to sign their section of the consent form and then it also automatically sends a copy of the consent form to the patient and to the practise. So, it automatically means that thinking about that good clinical practise the patients gets it, the practise gets it and us as a study team have that as a central repository so it makes it a lot easier for patient to consent… GP's obviously like that nature of being able to send the consent form out and then just being notified to say it's complete as well".*
Other benefits include the ability to send out follow up questions in different languages.	*"We're able to use REDCap to automatically send out our follow up questionnaires. So, every six months we send quality of life questionnaires that again, patients are able to do on their phone, they're able to do in different languages. And they're able to then that would so complete that automatically comes back us and saves in the REDCap database… it's able to be used by patients and we've always had good response and patients have found it easy to complete and we get really good sort of follow up questionnaire response rate. I think we've got very few that haven't completed their follow up questionnaires"*

For peer review only

Standards for Reporting Qualitative Research (SRQR)*

http://www.equator-network.org/reporting-guidelines/srqr/

Page/line no(s).

Title and abstract

**Title** - Concise description of the nature and topic of the study Identifying the study as qualitative or indicating the approach (e.g., ethnography, grounded theory) or data collection methods (e.g., interview, focus group) is recommended	1
**Abstract** - Summary of key elements of the study using the abstract format of the intended publication; typically includes background, purpose, methods, results, and conclusions	1-2

Introduction

**Problem formulation** - Description and significance of the problem/phenomenon studied; review of relevant theory and empirical work; problem statement	Pg3, L9-19
**Purpose or research questio**n - Purpose of the study and specific objectives or questions	Pg4 L1-4

Methods

**Qualitative approach and research paradigm** - Qualitative approach (e.g., ethnography, grounded theory, case study, phenomenology, narrative research) and guiding theory if appropriate; identifying the research paradigm (e.g., postpositivist, constructivist/ interpretivist) is also recommended; rationale**	Pg7 L15-16
**Researcher characteristics and reflexivity** - Researchers' characteristics that may influence the research, including personal attributes, qualifications/experience, relationship with participants, assumptions, and/or presuppositions; potential or actual interaction between researchers' characteristics and the research questions, approach, methods, results, and/or transferability	Not Included
**Context** - Setting/site and salient contextual factors; rationale**	P7 L16-20
**Sampling strategy** - How and why research participants, documents, or events were selected; criteria for deciding when no further sampling was necessary (e.g., sampling saturation); rationale**	P6 L24-25
**Ethical issues pertaining to human subjects** - Documentation of approval by an appropriate ethics review board and participant consent, or explanation for lack thereof; other confidentiality and data security issues	P26, L29-31
**Data collection methods** - Types of data collected; details of data collection procedures including (as appropriate) start and stop dates of data collection and analysis, iterative process, triangulation of sources/methods, and modification of procedures in response to evolving study findings; rationale**	P7 L1-20

For peer review only - http://bmjopen.bmj.com/site/about/guidelines.xhtml
For peer review only

**Data collection instruments and technologies** - Description of instruments (e.g., interview guides, questionnaires) and devices (e.g., audio recorders) used for data collection; if/how the instrument(s) changed over the course of the study	P7 L4-10
**Units of study** - Number and relevant characteristics of participants, documents, or events included in the study; level of participation (could be reported in results)	P4 Table 1
**Data processing** - Methods for processing data prior to and during analysis, including transcription, data entry, data management and security, verification of data integrity, data coding, and anonymization/de-identification of excerpts	P15 L3-9
**Data analysis** - Process by which inferences, themes, etc., were identified and developed, including the researchers involved in data analysis; usually references a specific paradigm or approach; rationale**	P7 L15-16
**Techniques to enhance trustworthiness** - Techniques to enhance trustworthiness and credibility of data analysis (e.g., member checking, audit trail, triangulation); rationale**	P7 L16-19

Results/findings

**Synthesis and interpretation** - Main findings (e.g., interpretations, inferences, and themes); might include development of a theory or model, or integration with prior research or theory	P15 – P22
**Links to empirical data** - Evidence (e.g., quotes, field notes, text excerpts, photographs) to substantiate analytic findings	P15 Table 5 + Supplementary Information

Discussion

**Integration with prior work, implications, transferability, and contribution(s) to the field -** Short summary of main findings; explanation of how findings and conclusions connect to, support, elaborate on, or challenge conclusions of earlier scholarship; discussion of scope of application/generalizability; identification of unique contribution(s) to scholarship in a discipline or field	P22-24
**Limitations** - Trustworthiness and limitations of findings	P24, L14-25

Other

**Conflicts of interest** - Potential sources of influence or perceived influence on study conduct and conclusions; how these were managed	P26 L21-25
**Funding** - Sources of funding and other support; role of funders in data collection, interpretation, and reporting	P26 L17-19

*The authors created the SRQR by searching the literature to identify guidelines, reporting standards, and critical appraisal criteria for qualitative research; reviewing the reference lists of retrieved sources; and contacting experts to gain feedback. The SRQR aims to improve the transparency of all aspects of qualitative research by providing clear standards for reporting qualitative research.

For peer review only

**The rationale should briefly discuss the justification for choosing that theory, approach, method, or technique rather than other options available, the assumptions and limitations implicit in those choices, and how those choices influence study conclusions and transferability. As appropriate, the rationale for several items might be discussed together.

Reference:

O'Brien BC, Harris IB, Beckman TJ, Reed DA, Cook DA. **Standards for reporting qualitative research: a synthesis of recommendations.** *Academic Medicine*, Vol. 89, No. 9 / Sept 2014 DOI: 10.1097/ACM.0000000000000388

For peer review only

Using Digital Tools in Clinical, Health and Social Care Research: A Mixed-Methods Study of UK Stakeholders

Journal:	*BMJ Open*
Manuscript ID	bmjopen-2023-076613.R1
Article Type:	Original research
Date Submitted by the Author:	13-Sep-2023
Complete List of Authors:	Clohessy, Sophie; University of Warwick, WMG<br>Arvanitis, Theodoros; University of Birmingham<br>Rashid, Umer; University of Warwick, WMG<br>Craddock, Carly; NIHR CRN WM<br>Evans, Mark; NIHR CRN WM<br>Toro, Carla; University of Warwick, Warwick Medical School<br>Elliott, Mark; University of Warwick, WMG
**Primary Subject Heading**:	Research methods
Secondary Subject Heading:	Qualitative research
Keywords:	Clinical Trial, QUALITATIVE RESEARCH, Telemedicine < BIOTECHNOLOGY & BIOINFORMATICS

SCHOLARONE™
Manuscripts

I, the Submitting Author has the right to grant and does grant on behalf of all authors of the Work (as defined in the below author licence), an exclusive licence and/or a non-exclusive licence for contributions from authors who are: i) UK Crown employees; ii) where BMJ has agreed a CC-BY licence shall apply, and/or iii) in accordance with the terms applicable for US Federal Government officers or employees acting as part of their official duties; on a worldwide, perpetual, irrevocable, royalty-free basis to BMJ Publishing Group Ltd ("BMJ") its licensees and where the relevant Journal is co-owned by BMJ to the co-owners of the Journal, to publish the Work in this journal and any other BMJ products and to exploit all rights, as set out in our licence.

The Submitting Author accepts and understands that any supply made under these terms is made by BMJ to the Submitting Author unless you are acting as an employee on behalf of your employer or a postgraduate student of an affiliated institution which is paying any applicable article publishing charge ("APC") for Open Access articles. Where the Submitting Author wishes to make the Work available on an Open Access basis (and intends to pay the relevant APC), the terms of reuse of such Open Access shall be governed by a Creative Commons licence – details of these licences and which Creative Commons licence will apply to this Work are set out in our licence referred to above.

Other than as permitted in any relevant BMJ Author's Self Archiving Policies, I confirm this Work has not been accepted for publication elsewhere, is not being considered for publication elsewhere and does not duplicate material already published. I confirm all authors consent to publication of this Work and authorise the granting of this licence.

For peer review only

BMJ Open- Revision 1 SC

Using Digital Tools in Clinical, Health and Social Care Research: A Mixed-Methods Study of UK Stakeholders

Sophie Clohessy[1], Theodoros N. Arvanitis[2], Umer Rashid[1], Carly Craddock[3,4], Mark Evans[3,4], Carla Toro[5], Mark T. Elliott[1]

[1]Institute of Digital Healthcare, WMG, University of Warwick, Coventry, United Kingdom

[2]School of Engineering, University of Birmingham, Birmingham B15 2TT, UK

[3]The Royal Wolverhampton NHS Trust, Wolverhampton, UK

[4]National Institute for Health Research Clinical Research Network West Midlands, Birmingham, UK

[5]Division of Health Sciences, Mental Health and Wellbeing Group, Warwick Medical School, University of Warwick, Coventry, United Kingdom

Corresponding author's contact details: Mark T. Elliott, email: m.t.elliott@warwick.ac.uk

Word counts

Abstract:315

Main text: 5200

Abstract

Objective: The COVID-19 pandemic accelerated changes to clinical research methodology, with clinical studies being carried out via online/remote means. This mixed-methods study aimed to identify which digital tools are currently used across all stages of clinical research by stakeholders in clinical, health and social care research and investigate their experience using digital tools.

Design: Two online surveys followed by semi-structured interviews were conducted. Interviews were audio recorded, transcribed, and analysed thematically.

For peer review only - http://bmjopen.bmj.com/site/about/guidelines.xhtml

Setting, Participants To explore the digital tools used since the pandemic, survey participants [Researchers and Related Staff (n=41), Research and Development staff (n=25)], needed to have worked on clinical, health or social care research studies over the past two years (2020-2022) in an employing organisation based in the West Midlands region of England (due to funding from a regional clinical research network). Survey participants had the opportunity to participate in an online qualitative interview to explore their experiences of digital tools in greater depth (n=8).

Results: Six themes were identified in the qualitative interviews: '*Definition of a Digital Tool in Clinical Research*'; '*Impact of the COVID-19 Pandemic*'; '*Perceived Benefits/Drawbacks of Digital Tools*'; '*Selection of a Digital Tool'; 'Barriers and Overcoming Barriers';* and '*Future Digital Tool Use'.* The context of each theme is discussed, based on the interview results.

Conclusions: Findings demonstrate how digital tools are becoming embedded in clinical research, as well as the breadth of tools used across different research stages. The majority of participants viewed the tools positively, noting their ability to enhance research efficiency. Several considerations were highlighted; concerns about digital exclusion; need for collaboration with digital expertise/clinical staff, research on tool effectiveness and recommendations to aid future tool selection. There is a need for the development of resources to help optimise the selection and use of appropriate digital tools for clinical research staff and participants.

Keywords

Digital Tool, Clinical Research, Clinical Trial, Qualitative, Survey, COVID-19

Article Summary

Strengths and Limitations of the Study

For peer review only

For peer review only - http://bmjopen.bmj.com/site/about/guidelines.xhtml

- Mixed-method study to explore experiences of utilising digital tools in clinical research, utilising participants from a variety of roles; both Research and Research and Development staff.
- Survey and qualitative questions co-designed with an experienced stakeholder group.
- It is likely that participants were motivated to participate due to an underlying interest in digital tools, consequently some perspectives may not have been included in this study.
- The study was limited to exploring the views of stakeholders employed by organisations based in the West Midlands, United Kingdom.

Introduction

Digital tools are increasingly used across stages of clinical research from recruitment (e.g., social media platforms) to data collection (e.g., online survey platforms, online workshops) (e.g., 1, 2-4), with the applicability of digital tools within clinical research ever widening . Digital tools can be defined as an alternative to paper-based methods that is IT based or an online platform that aids any aspect of the research study. The COVID-19 pandemic accelerated changes to clinical research methodology, social distancing forced study teams to transition from conducting research activities in person to predominantly online and/or via remote means (e.g., 1, 5, 6). Documented challenges associated with rapid adoption of digital tools in clinical research include; staff/participant lack of familiarity with or limited access to digital tools and rapid changes to protocols/ethics applications to ensure compliance (5).

A recent report by the UK government [see *'The Future of Clinical Research Delivery: 2022 to 2025 implementation plan*'] sets out a future vision of research delivery which includes data enabled trials (i.e., using electronic health records) and digital tools (7). Given that digital tools are now commonplace and set to be firmly embedded within clinical research, there is a need to explore people's experiences using digital tools; what has worked well and not so well in order to understand

For peer review only - http://bmjopen.bmj.com/site/about/guidelines.xhtml

their value, relevance and use in future clinical research. A recent mixed method study by Blatch-Jones, Nuttall (8) explored which digital tools were used by stakeholders within recruitment and retention in clinical research in the UK as well as investigating experiences using these tools. Whilst previous research has investigated UK stakeholder's opinions on digital tool adoption for recruitment and retention, this has left other research stages understudied. It is evident there is a need to explore experiences of digital tools across the entire breadth of research stages (e.g., informed consent, set up, data collection, intervention delivery, management of research studies) as well as investigating the impact of the COVID-19 pandemic on the switch to digital tools for facilitating clinical research. Indeed, a particular focus of this study was on the use and experience of digital tools in the context of the rapid switch to digital methods during the COVID-19 pandemic in the UK between 2020 and 2022.

The aim of this study was to explore examples of digital tools used by stakeholders across the West Midlands geographical region in clinical, health and social care research.

Methods

This study comprised of two main components: *Phase 1:* Online surveys to understand the overall picture of digital tool adoption across the region, with two key stakeholder groups: 1) Researchers and Related Staff 2) Research and Development [R&D] Staff 3) Participants and Carers[1] (see Table 1, for the definition of these groups). *Phase 2:* Online survey participants were given the opportunity to participate in qualitative interviews to build upon survey answers, exploring their experiences with digital tools in further depth. The survey and qualitative questions were co-designed with an experienced stakeholder group consisting of three individuals working in senior positions within

[1] A third online survey was distributed to participants and carers to understand their experience when using digital tools. However due to limited participant responses (n=4), no data analysis took place.

For peer review only - http://bmjopen.bmj.com/site/about/guidelines.xhtml

clinical research based in the West Midlands. The stakeholder group met once during the project on Microsoft Teams (February 2022), the research team presented an overview of the project and survey questions and the steering group offered verbal feedback. The stakeholder group members were emailed a draft of qualitative interview materials in August 2022 and provided feedback via email on the qualitative questions.

Patient and Public Involvement No patients and/or public were involved in the design or conduct or reporting or dissemination plans of this research.

Patient consent for publication Not required.

Phase 1: Online surveys

Participants

Two surveys were aimed at the following participant groups, with eligibility criteria shown in Table 1.

Table 1: Eligibility criteria by survey type

Survey Type	Participant Group	Eligibility Criteria
Researchers and Related Staff	People working on clinical research projects either in a research capacity or operational support.	Aged 18 years or older; employing organisation based in West Midlands region of the UK; working on clinical research projects over the past two years (2020-2022)
Research & Development (R&D) Staff	People working within a hospital's Research & Development (or similar) department, with a role to support or coordinate clinical research taking place within, or in partnership with the Hospital.	Aged 18 years or older; hospital must be a research active NHS Trust in the West Midlands region of the UK; working in a hospital's Research & Development (or similar) department over the past two years (2020-2022)

Recruitment

i) **Researchers and Related Staff Survey.** Participants were recruited primarily using databases of clinical, health and social care research studies held by the National Institute of Health Research Clinical Research Network[2] - NIHR CRN West Midlands. An initial list of relevant projects were obtained by NIHR staff using the Open Data Platform (ODP) that

[2] NIHR, a virtual organisation, is the research arm of the National Health Service. The NIHR Clinical Research Network (CRN) is made up of 15 local networks across England which helps patients, the public and health and care organisations to participate in high-quality research

For peer review only - http://bmjopen.bmj.com/site/about/guidelines.xhtml

transforms the data held in the NIHR's Central Portfolio Management System (CPMS) into a usable form, allowing it to be tabled, filtered, and organised by various metrics. Projects were selected based on the following criteria: Clinical, health or social care projects led by the Clinical Research Network West Midlands, that had been completed or were ongoing within the previous three years[3] (with any recruitment activity during the four financial years 2019/20 - 2022/23, filtered to include only those studies opened since 01/06/2019). Additional data was sourced from CPMS and the Local Portfolio Management System (LPMS), which functions as a secure data storage platform for the records of study approvals, delivery & site activity at a regional level. An email invitation and survey link were then distributed to the lead researcher or main contact for the selected research studies (approximately 400 contacts in total).

ii) **R&D survey.** A total of 26 NHS Trust R&D departments in the West Midlands were contacted by NIHR staff via email invitation and survey link.

For both surveys, to further widen the opportunity to participate, a short study description and survey links were advertised on Twitter via the NIHR CRN West Midlands account. After a period of 2 weeks a follow-up reminder email was sent to all contacts via the same mechanism as the initial survey invitation. Both surveys were live for one month in total. We anticipated approximately 50 people would complete each survey, however after the first data collection period (27th June - 26th July 2022), participant numbers were lower than expected (35 for Research and Related Staff survey and 15 for R&D); therefore, a second phase of recruitment took place (14th October - 15th November 2022). An email prompt was sent to all contacts via the same mechanism as the initial survey invitation.

Procedure

[3] Although, we were primarily interested in including studies which had used digital tools between the years 2020-22 (since beginning of COVID-19), studies were included from the past three years (2019-2022) to ensure a suitable number of studies were included in the survey. Most research was paused to deliver urgent public health research during the pandemic, and then possibly restarted during the two-year window.

For peer review only - http://bmjopen.bmj.com/site/about/guidelines.xhtml

Participants accessed surveys via a hyperlink. The surveys were created and hosted on Qualtrics, a secure online survey platform (https://www.qualtrics.com/uk/), which is used for creating surveys with in-depth response formats and allows for sophisticated methods of distribution and data management (9). Before taking part, all participants read an information sheet and ticked a consent form (embedded into the Qualtrics platform) to agree to their participation, after survey completion, participants were presented with a debriefing sheet which repeated the objectives of the study and information about how the survey data would be stored. Before answering any questions, participants were offered a definition of a digital tool and examples of digital tools (See Supplementary Materials 1, Table S1). The definition varied between the two surveys to account for tools used in a variety of contexts. All survey participants were invited to participate in a qualitative interview study to explore their thoughts towards digital tools in further detail.

i. Researchers and Related Staff Survey: Procedure Overview

Participants were re-directed to the end of the survey if they did not meet the eligibility criteria (see Table 1) determined via initial screening questions. A further screening question determined if participants had used digital tools in their research studies. Participants who answered 'yes' to using digital tools in their clinical, health or social care research were then directed to questions which focused on participants' experience of what they perceived to be most effective and least effective digital tools used in their role (blocks presented in a randomised order, questions were the same in each block) (See Supplementary Materials 1, Table S2 for question themes and Supplementary Materials 2a for Online Survey Questions).

ii. R&D Staff Survey: Procedure Overview

Eligible participants answered three blocks of questions about their perceived most effective digital tool used in 1) Set up 2) Recruitment and 3) Management of Clinical Research Studies (blocks were presented in a randomised order, questions were the same in each block). If participants did not have

For peer review only - http://bmjopen.bmj.com/site/about/guidelines.xhtml

a digital tool example for set up, recruitment or management they skipped this block of questions and were re-directed to the next part of the survey (See Supplementary Materials 1, Table S2 for question themes and Supplementary Materials 2b for Online Survey Questions).

Data Analysis

A mixed approach to analysis was undertaken for both surveys. Multiple choice and scale answers were analysed using frequencies and percentages.; free text answers were analysed thematically. Results from both surveys were analysed, discussed, and verified with the project team.

Phase 2: Qualitative Interviews

Recruitment

During the online survey, participants were invited to take part in the qualitative interviews and expressed their interest by providing their name, job role and email address.

Procedure

All interested participants were sent an electronic information and consent sheet via email and a mutually convenient time and date was arranged via Doodle poll (https://doodle.com/free-poll). Participants returned consent via email; this was also confirmed verbally at the beginning of each interview. Semi-structured interviews were undertaken by the Research Fellow (Author, SC) via Microsoft Teams, lasting between 45-60 minutes (conducted between 20th October- 9th November 2022). The interview topic guides (See Supplementary Materials 1, Table S3 for an overview and Supplementary Materials 3a and 3b for full interview questions) were informed by the online survey; participants were informed that their survey answers would be discussed in greater detail during the qualitative interviews (referenced on the consent form). All interviews were recorded and transcribed using the built in functionality in Microsoft Teams. Data collection was stopped at the point of data saturation; this was defined as the point of which no new themes were observed.

Data Analysis

Transcripts were anonymised and cross-checked for accuracy by the first author (SC) and Research Assistant (UR) against the recordings. Interview transcripts were then uploaded into NVivo qualitative analysis software (Version 12, QSR International, Massachusetts, US). A Thematic Analysis (10) was

For peer review only

applied, using an inductive approach, in accordance with Braun and Clarke's six-stage model. Transcripts were coded on NVivo by two raters (SC and UR). To reduce bias, a second rater (rater 2, UR) independently coded 25% of all interviews using the same method as rater 1 (SC). To enhance validity, following their independent assessments, the raters met to compare, contrast, and refine existing codes until agreement was reached on the grouping of main themes and subthemes. Themes and subthemes were then presented and demonstrated by representative quotes. All analyses were reviewed by the Primary Investigator and results were presented and discussed with the PI and Co-Investigators on a fortnightly basis.

Results

Phase 1: Online Survey Results

i. Research and Related Staff Survey Results

A total of 80 people read the information sheet and provided consent. 52/80 (65%) people completed the initial survey question; "Thinking about the clinical, health or social care research studies you are currently working on or have worked over the past two years, have you used digital tools to assist in the operation, management or coordination of these studies?" of which eight participants answered that they delegated tool use to colleagues and two participants answered 'no' to having used digital tools; one participant provided reasoning for not using digital tools which was 'stick to what worked in the past' (selected from drop-down menu of answers). These ten participants were re-directed to the end of the survey as the study was primarily interested in exploring experiences of people who had first-hand experience of using digital tools within their research. If participants only had experience of one digital tool, they were asked questions about this specific example. One participant answered questions about one digital example, however, did not provide the name of the digital tool so therefore this participant was removed from analysis as the tool could not be categorised under a research stage (e.g., participant recruitment).

For peer review only - http://bmjopen.bmj.com/site/about/guidelines.xhtml

For peer review only

1 Of those 41 participants partially or fully completed the survey (see Supplementary Materials 1, Table S4 for participant characteristics). As an introductory question, participants were asked to list all of the digital tools they have used over the past two years, 11/41 people completed the survey up until this point. Thirty participants continued and provided one digital tool example which they deemed to be 1) the least effective tool and/or 2) the most effective tool, with 19/30 participants answering a number of questions about these tools and completing the survey. Table 2 provides an overview of the most effective and least effective tools by research stage.

Table 2: Overview of most effective and least effective digital tools examples by research stage

**Research Stage**	**Most Effective Examples**	**Least Effective Examples**
Data collection	Castor EDC<br>Electronic completion of questionnaires<br>Electronic database to collect participant data from participating hospitals.<br>MS Teams<br>Online data capture tool<br>Patient Identification Centre (PICS)<br>REDCap tool to upload patient data<br>Teams/Zoom for participant interviews<br>Text messaging	Trial Database<br>Randomisation<br>Database for collected intervention information
Intervention delivery	Smart survey<br>Trial intervention delivered to participants via online web portal	Digital intervention as it requires hardware to be used by the patient/research team
Other	MS Teams	MS Teams
Outcome measures	In house databases	n/a
Participant recruitment	QR code for accessing online survey<br>Social media to recruit participants<br>REDCap e-consent system enables GP practices to remotely consent patients to take part in the trial<br>Twitter page for trial<br>Using an online platform to host consent/demographic forms	Text messaging to invite participants to take part in a survey<br>Facebook and Twitter<br>Asking GP practices to use their SMS systems for inviting patients to questionnaire studies.<br>Health Survey<br>Email - to participants<br>SMS [Text Message]<br>Social media for recruitment<br>Advertising the study's recruitment advert on reputable charities social

For peer review only

		media accounts<br>Clinical Practice Research Datalink Interventional Research Services Platform system<br>Reports set up to highlight which participants have dropped out in between expressing interest and being randomised.
Research study set up	REDCap<br>WhatsApp<br>MS Teams	Teams<br>Online conferencing tools (dependent on the good connection of all participants)<br>Investigator site file templates distributed via online file sharing service
Quality assurance	[none listed]	Datix
Stage not provided	Castor EDC for sending out surveys to be completed online<br>Remote research meetings with site collaborators	[none listed]

Most tools referenced were from ongoing clinical trials, with the largest number of digital tools referenced from data collection (most effective digital tools) and participant recruitment (least effective). Most tools were widely available, as opposed to a bespoke tool. Notably, some tools (e.g., social media, Microsoft Teams) were listed as both Most Effective and Least Effective tools, highlighting the variation in experiences when using these platforms. Table S5 in Supplementary Materials 1 provides a breakdown of participants' experiences of using the most and least effective tools they listed, including training requirements and knowledge of costs. Participants selected a range of 1-5 goals for using digital tools, of which the most popular research goal for using digital tool was "Reduce time/Increase efficiency". The most common users of digital tools referenced by participants were research participants (most effective digital tools) and research delivery (least effective digital tools).

For peer review only - http://bmjopen.bmj.com/site/about/guidelines.xhtml

In context of most effective tools, on a scale of 0 - 10 (0 no more effective than other tools to 10 much more effective than other tools) participants gave a mean rating of 8.1±1.3 (see Table S6, supplementary materials 1). This result suggests that participants were mostly positive about the tools they deemed to be efficient. In contrast, the least effective tools, were rated on a scale of 0 - 10 (0 = no less effective than other tools, 10 = much less effective than other tools) with a mean rating of 4.3±2.0. see Table S7, supplementary materials 1).

For peer review only

ii. Research and Development Survey Results

A total of 35 people accessed the survey link and completed the consent form. A total of 25/35 (71.4%) people completed the introductory question 'Has your department used digital tools in the set-up, recruitment and management/monitoring of clinical research? (see Supplementary Materials 1 Table S8 for participant characteristics) of which three participants answered 'no' to having used digital tools; three participants provided reasoning for not using digital tools (selected from a drop-down menu of answers). 22/35 (62.9%) participants went onto partially or fully completed the survey.

The largest number of most effective tool examples, provided by the R&D survey respondents were for management of clinical studies (N=11). However, the tool referenced in this, and the categories varied substantially, i.e., in most cases, each tool was only mentioned by one participant (see Table 3). Table 4 provides a breakdown of participants' experiences of using the most and least effective tools they listed, including training requirements and knowledge of costs.

Table 3: Most effective digital tools examples by research stage used by employees working in R&D

Stage of Research	Most Effective Digital Tool Example	N
Set Up	Excel Trackers	1 (25%)
	Informatics	1 (25%)
	Power BI	1 (25%)
	SharePoint	1 (25%)
		4
Recruitment	Social Media platforms	4 (66.7%)
	PICS	1 (16.7%)
	Contact for Research Database	1 (16.7%)
		6 (16.7%)
Management	Electronic signing of contracts	1 (9.05%)
	Power BI	1 (9.05%)
	REDCap	1 (9.05%)
	TriNetX	1 (9.05%)
	DocuSign	1 (9.05%)
	Audit	1 (9.05%)
	Study Tracker	1 (9.05%)
	MS Teams (data collection)	1 (9.05%)
	Online Case Report Form	1 (9.05%)
	Patient Trackers	2 (18.2%)
		11

For peer review only

For peer review only

For peer review only - http://bmjopen.bmj.com/site/about/guidelines.xhtml

Table 4: R&D staff summary of digital tool use

Question Type	Question Responses	Set Up N (%)	Recruitment N (%)	Management N (%)
Total number of most effective examples	n/a	4	6	11
How did they hear about digital tool	Developed in house	1 (25%)	3 (50%)	4 (36.4%)
	Other	1 (25%)	2 (33.3%)	2 (18.2%)
	Recommended by others	2 (50%)	n/a	2 (18.2%)
	Saw advertised online/social media	n/a	1 (16.7%)	n/a
	Approached by digital tool provider	n/a	n/a	3 (27.2%)
	Total responses	4	6	11
Training required for digital tool	Yes	2 (50%)	2 (33.3%)	7 (63.4%)
	No	2 (50%)	4 (66.6%)	4 (36.6%)
	Total responses	4	6	11
If yes to training, training length	< 1 day	1 (50%)	1 (50%)	4 (57.1%)
	1-5 days	n/a	1 (50%)	n/a
	> 5 days	1 (50%)	n/a	3 (42.9%)
	Total responses	2	2	7
Widely used or bespoke digital tool	Widely used	4 (100%)	5 (83.3%)	8 (72.7%)
	Novel/Bespoke to organisation	n/a	1 (16.7%)	2 (18.2%)
	Novel/bespoke to project team	n/a	n/a	1 (9.1%)
	Total responses	4	6	11
Costs associated with acquiring digital tool	I don't know the answer	1 (25%)	1 (25%)	2 (25%)
	No fee (e.g., open source)	1 (25%)	2 (50%)	2 (25%)
	Periodic recurring fee	1 (25%)	n/a	2 (25%)
	Other	1 (25%)	1 (25%)	2 (25%)
	Total responses	4	4	8

For peer review only

For peer review only - http://bmjopen.bmj.com/site/about/guidelines.xhtml

Cost effectiveness (1 highly ineffective to 7 highly effective)	3	1 (25%)	n/a	n/a
	4	1 (25%)	n/a	n/a
	5	1 (25%)	4 (100%)	n/a
	6	n/a	n/a	4 (50%)
	7	1 (25%)	n/a	4 (50%)
Total responses		4	4	8

For peer review only
For peer review only - http://bmjopen.bmj.com/site/about/guidelines.xhtml

BMJ Open- Revision 1 SC

Phase 2 Results: Qualitative Interviews

A total of eight participants took part in the interviews; five participants had completed the survey for R&D employees, three participants had completed the Researchers and Related Staff survey. Participants were employed by a variety of organisations in the West Midlands area. Six main themes were identified across the transcripts, these are summarised in Table 5 along with selected quotes relating to each theme, discussed in detail below. Two case studies of most effective tools have been summarised in Table S9, supplementary materials 1; Case Study 1 (Management) and Case Study 2 (Recruitment).

For peer review only - http://bmjopen.bmj.com/site/about/guidelines.xhtml

Table 5: Summary of identified themes and representative quotes

Theme	Sub Theme	Example Statements
1. Definition of Digital Tools in Clinical Research	n/a	*[1.1] "Anything that you can use online basically. So, it can be a handset, it can be a tablet or a website…it's anything that can connect to the Internet that can ease the process of undertaking research."*<br>*[1.2]"It helps make it easier to collect data…easier to track where research is […] And then thinking about the patient facing role of digital research…giving devices to patients or using patients own devices to actually report outcome measures directly to research teams…using technology, whether it's Internet based technology or sort of phone, tablet, laptop-based technology to hopefully improve research even like an e site file system or a trial master file or trial master file system or just using teams and SharePoint, this is a digital solution."*
2. COVID-19 Pandemic: Validating the benefits of working digitally	n/a	*[2.1]"We wanted to continue the research where we could do so…we had to move it online. And people just embraced that. We had our IT team. We had enough resources to get everything onto online platforms in order to collect the data on an online questionnaire as opposed to a paper questionnaire."*<br>*[2.2]"We proved that the digital way is working, so that's massive. It definitely gave us the push needed cause we had no choice. People had to start using digital tools and actually once we got through it (Covid) […] you go actually, we're not gonna go back to that way before because this way works really well…and it gave us a lot more buy in, particularly with some nurses who may be resistant to change."*
3. Perceived Benefits/Drawbacks	3a. Perceived Benefits	*[3.1] "For the patients that are happy and IT savvy and things it saves a trip to the post box or whatever to fill in the paper questionnaire if people are happy to do it, especially because it's like transferable, you can answer it on your whether it be a mobile phone or a tablet or a PC."*<br>*[3.2]"We potentially can reach patient populations that wouldn't normally take part in research, those that potentially have a lower mobility, aren't able to travel as much could actually take part in the trial."*<br>*[3.3]"You can ask people not only to talk but also to use the chat, which I think helps some people who are bit kind of less able to interact in a group."*<br>*[3.4]"It's easier on an online tool to have a number of different languages …if you're printing off something that's a hell of a lot of paper…and you're sort of sometimes guessing what language people are wanting things in. If there's a drop-down box that could all of a sudden change it to French or you know Arabic, it's a much easier thing to be able to do."*<br>*[3.5]"WhatsApp has been used to keep contact with researchers between meetings, which I felt was quite effective…so it helps to have a WhatsApp group and say what are you doing? Can I help? It helps between meetings to keep things going."*
	3b. Drawbacks	*[3.6]"In my experience online is good to get a lot of work done rapidly but sometimes you can't read face to face interactions. You can create maybe bonds that are bit beyond the business like you can have a coffee together so there's a little bit of a difference there."*<br>*[3.7]"Some participants might struggle with connection and so on. So, there's a number of technical issues that [could occur] but again that's very similar to a logistical issue that you would have in a face-to-face workshop."*<br>*[3.8]"The further negatives are probably people having to use a new system if they don't use it that often then potentially forgetting how to use it. But obviously we've tried to … reduce that by offering videos, offering…training sessions."*<br>*[3.9]"I think drawbacks are still engagement and I think this is what we're trying to investigate at the moment… what is engagement like across different participant groups and age ranges and demographics and ethnicities and stuff like that… I'm not sure it is inclusive."*
4. Selection of a Digital Tool and Recommendations for Future Digital Tool Use	4a. Selection	*[4.1]"It was basically chosen because it was free, and that's what the university had".*<br>*[4.2]"Things tend to be sort of by word of mouth I think a lot relies upon the fact that you happen to have a conversation you know when somebody says ohh, hold on a minute you could do it this way or you could use this."*
	4b. Recommendations	*[4.3]"Almost a digital checklist and then it points to what program, even if it doesn't say what the program is, it tells you the type of program you need…depending on where you are with it and what you're doing. Are you the sponsor? Well, if you sponsor a study you'll need a very different [tool] to if you're delivering the study… is it multi-site?."*<br>*[4.4]"I think it would be good to have case studies of people who have used some tools and made their own adaptations [for a] service user or participant group."*<br>*[4.5]"If you don't know what's available you, you don't know what you don't know. There could be a digital solution to the problem you've got but if that's not*

For peer review only - http://bmjopen.bmj.com/site/about/guidelines.xhtml

BMJ Open- Revision 1 SC

		your background, if you haven't encountered it, you wouldn't know to search for it.... I think a checklist...suggestions or case studies essentially and almost you know sharing best practice across regions…It could be national."<br>*[4.6]"A community of practice might be helpful because then you can bring people together […] and […] exchange information. So, I guess developing a network of practitioners working [on] research using tools… that might help to improve the way we use them."*
5. Barriers and Overcoming Barriers	5a. Barriers	*[5.1]"It feels like people were happy to make the move because you couldn't [use] paper. But following COVID some people have gone back to paper…especially older people […] they said…that they'd be worried about clicking on a link and whether it was, you know, bonafide or not…whether it is safe to click on the link."*<br>*[5.2]"Digital tools are there for everyone. Some teams grab with them and run with them really quickly. Others are really resistant still and I think it's what you know. And it's not clear cut on age at all, but there definitely is a demographic that can feel more comfortable with it, and others that don't and I think that comes down to experience."*<br>*[5.3]"They are trained as nurses first and foremost[…]their first priority is always gonna be their patients and you know, hear stories about them working super long shifts, they don't take a break for the entire time, when are they gonna have time to sit down and play with Excel."*<br>*[5.4]"If they've got no budget for IT, then they'll need to whip up an Excel spreadsheet."*<br>*[5.5]"We've got ideas as to what would make life easier for us, which ultimately makes life easier for staff and for patients […] we don't have the expertise behind it so we would be expecting other people to do it. So, the barrier really is sometimes the experience of putting your idea into something that will actually work. This cost is always going to be a barrier in the NHS…if we don't invest in something, how are we ever going to move forward… and so if we invest in there, then actually we might be able to start, you know, save on staff time. So, I think the biggest barrier is expertise."*
	5b. Overcoming Barriers	*[5.6]"I did a questionnaire study and used 18 practices…I sent a pre-notification text to nine practises and nine practices I just sent the link to the study and there was absolutely no difference in participation rate."*<br><br>*[5.7]"We keep an open mind as to whether people want to show their face or not. Because some people are not comfortable with showing their house, you know it's quite invasive in a way […] if some people want to keep the cameras on, we say we prefer them to keep the camera on. But if they want to keep off and just participate by audio [we're] also quite open to that."*<br><br>*[5.8]"There's a potential that maybe people who aren't technologically aware would struggle to take part in the study […] it isn't an all or nothing because this is a remote study it has to be remote, no you can actually take that remote nature away and just have a patient complete it on a practice computer."*
6. Future Digital Tool Use	n/a	*[6.1]"Maybe in 20 years and everybody will really use them. So digital exclusion might reduce over time I feel."*<br>*[6.2] "Clinical trials seem to be becoming more complex […] I think people are more inclined to look at digital support with studies […] I think historically […] nurses would be expected to pick up these sorts of administrative tasks. I think now we're looking at getting people in who can do it and it's a lot more efficient."*<br>*[6.3]"Researchers here come and say to me…what's your evidence for using it? What's are your response rates for those via paper and those that answered via e-mail or text message? […] I just think that needs to be published a bit more so people can make informed decisions around what they're going to use for their particular sample."*

For peer review only - http://bmjopen.bmj.com/site/about/guidelines.xhtml

BMJ Open- Revision 1 SC

Theme 1: Definition of Digital Tools in Clinical Research

Participants perceived a digital tool used within clinical research as a device that can connect to the internet, or a form of online technology. The main reasoning cited for using a digital tool was to increase efficiency within research (Table 5, Quotes 1.1-1.2). Digital tools have been adopted across all stages of clinical research from recruitment to data collection, it was noted that tools may differ depending on the research stage and/or role (e.g., R&D staff may encounter different digital tools compared to research participants).

Theme 2: COVID-19 Pandemic: Validating the benefits of working digitally

It is evident that the COVID-19 pandemic accelerated the use of digital tools within clinical research. It was noted how changes to research methodology needed to be implemented quickly; moving from paper-based methods to digital methods. COVID-19 was perceived as a time to illustrate the benefits of digital tools which helped to alter stakeholder's views who may have been pessimistic towards tools prior to the pandemic. Despite changes in working practices, participants perceived attitudes among staff and research participants to be mostly positive towards tools. The pandemic served as a trial period, to prove that *"the digital way is working"*, supporting continued digital tool use and innovation in routine practise. There was a sense of resistance from participants to not revert back to seemingly less efficient non digital methods (i.e. paper questionnaires compared to online).

Theme 3: Perceived Benefits and Drawbacks of Digital Tools

3.1. Benefits

Benefits of tools were considered from two perspectives; research participants and staff using digital tools. For research participants, perceived benefits of using tools included convenience and increased opportunities to take part in research. It was also noted that digital tools can account for individual differences of research participants; for example, some research participants may feel more

For peer review only - http://bmjopen.bmj.com/site/about/guidelines.xhtml

comfortable contributing to a focus group using the chat function on Microsoft Teams rather than speaking aloud. From the perspective of staff, interview participants considered efficiency (e.g., clinical delivery staff spending less time on admin tasks leading to more time with trial participants), easy connectivity between colleagues (particularly if based in different locations) and ease of offering research materials in different languages as primary benefits of digital tools (see Table S9, Case Study 2 for most effective digital tool for recruitment). (Table 5, quotes 3.1-3.5).

3.2. Drawbacks

Some of the drawbacks of digital tools referenced were of a practical nature, for example technical issues such as poor internet connection or researchers may require additional technical support. However, it was noted that face-to-face research studies may also require additional support. Other drawbacks related to connection, specifically difficulty reading interactions online. Despite the ease of meeting with colleagues online, especially when based in different locations and organisations, there was some concern that meeting colleagues online does not always enable people to strengthen or develop relationships in the same way as meeting face to face allows. Participants raised concerns around inequalities and lack of inclusivity relating to digital tool use, it was noted that levels of engagement are currently unclear from different research participant groups when using various digital tools. Digital literacy was identified as a potential risk to excluding certain people with a lack of familiarity with using digital tools, especially if research studies are conducted solely using digital tools (Table 5, quotes 3.6-3.9).

Theme 4: Selection of a Digital Tool and Recommendations for Future Digital Tool Use

4.1. Selection

Most participants cited having little choice in the digital tool they used, this was mainly due to the fact that most tool examples were widely used in an organisation (e.g., Microsoft Teams) and as a result they did not have the authority to select a tool. Despite this, it was recognised that familiarity with

For peer review only - http://bmjopen.bmj.com/site/about/guidelines.xhtml

widely used tools was beneficial for ease of use. There was a concern that often staff are not aware of what the most appropriate tool would be for a research task. Some bespoke tools were utilised; two participants described the process of designing and developing a bespoke digital tool in collaboration with clinical staff (see Table S9, case study 1 for most effective digital tool for management). The most common way staff discovered about digital tools appears to be through word of mouth (Table 5, quotes 4.1-4.2).

4.2. Recommendations for future tool selection

A number of recommendations were proposed to assist staff in future tool selection. There appears to be no standardised process outlining how to choose a tool, therefore a checklist database was proposed in which staff could input their digital tool requirements (e.g., research stage, user type) and a programme could provide appropriate digital tool options. Further recommendations included a central place (e.g., website) to share information about tools, case studies of effective tools (by research stage/stakeholder group) and a local/national network to share digital tool expertise (Table 5, quotes 4.3-4.6)

Theme 5: Barriers and Overcoming Barriers

5.1. Barriers

Several barriers to digital tool use were referenced including attitudes of staff and research participants; one participant explained that older research participants were concerned about the legitimacy of receiving a survey link via email. A commonly cited barrier was the conflict of carrying out a patient centred job (i.e., nurse) whilst finding time to use digital tools. Resources were also cited as a barrier to digital tool use; resources included budget, physical resources (e.g., poor internet connection or limited digital equipment), digital expertise (some organisations had access to specialist digital teams embedded in a Research and Development department) as well as digital literacy (research participants and staff). Available resources varied across organisations and research

For peer review only

participants; appearing to be both a key barrier as well as a key driver for digital tool use (Table 5, quotes 5.1-5.5).

5.2. Overcoming Barriers

A number of ways to overcome barriers were proposed by interview participants. Some reported providing research participants with a physical location to visit to complete research activities on a computer (if they did not have access to a computer and/or internet connection). Other participants reported using a hybrid approach of both paper and digital tools to carry out the same research tasks. A hybrid approach was undertaken to reduce digital exclusion, catering for research participants who may not have access to digital tools/internet and/or prefer paper-based methods. However, an impact of this approach included a larger workload due to increased data management.

It is evident that individual differences exist with digital tool use, with interview participants described adopting a flexible approach with research participants. For example, some research participants may not feel comfortable using their video camera when using Microsoft Teams. Interviewees explained how research participants and staff attitudes can be a barrier to digital tool use but offering training in multiple modalities can help to increase users' confidence (e.g., video, face-to-face demos). Some interviewees mentioned conducting their own research to evaluate the effectiveness of digital tools. For example, one participant had previously compared two recruitment methods; a text message in advance informing participants they would receive a survey link via email compared to no pre-notification at all. However, this study reported no differences in the number of participants recruited to the study via either method, suggesting that reassurance of the legitimacy of the survey link did not have any impact in terms of increasing engagement (Table 5, quotes 5.6-5.8).

Theme 6: Future Digital Tool Use

All participants perceived they would be using tools increasingly in the future and that digital exclusion of research participants may reduce over time due to increasing familiarity with digital tools.

For peer review only - http://bmjopen.bmj.com/site/about/guidelines.xhtml

Participants highlighted the potential for pressure to be placed on nurses to keep up to date with digital tools whilst caring for patients, as well as the need for collaboration between digital experts and clinical staff when developing and utilising tools. Suggestions for research to inform future tool use included investigating which tools are most effective for different research stages and stakeholder/participant groups and research exploring the cost effectiveness of digital versus paper-based tools. Further suggestions included organisations conducting training analysis of all stakeholders using digital tools (Table 5, quotes 6.1-6.3).

Discussion

This mixed-method study aimed to explore which tools are being used across clinical research as well as staff experiences of digital tool use. Our findings indicate that digital tools are now a fundamental part of clinical research; the COVID-19 pandemic accelerated the use of tools with adoption observed across all research stages (e.g., 1, 11-13). Tools were mostly viewed favourably, with increased efficiency cited as a leading benefit for research staff. It was noted that tools can account for individual differences of research participants, potentially offering research opportunities to a broader sample of participants (1, 8, 14, 15). For example, materials can be easily offered to research participants in different languages and reduce participants' need to travel to a physical location. Despite this, some drawbacks, and barriers to the use of tools exist. In particular, there were concerns about digital exclusion of research participants who may not have access to digital tools, and/or internet connections, or lack familiarity with using these platforms (e.g., 1, 16-18). There was also a concern about limited resources within clinical research; particularly the need for individuals with digital expertise to advise on and develop digital tools.

A previous mixed methods study by (8) explored which digital tools were being used for recruitment and retention within clinical trials. The current study extends this research by investigating UK stakeholders digital tool use in the context of the COVID-19 pandemic as well as across a wider range of research stages (i.e., informed consent, management, data collection),

For peer review only - http://bmjopen.bmj.com/site/about/guidelines.xhtml

considering not only the benefits, but also the limitations of digital tools. Consistent with the afore mentioned study by (8), our findings add to existing evidence that digital tools provide opportunities to widen reach of participation as well as reflecting previous concerns around digital exclusion for some groups (1, 8, 19, 20). New insights from the present study compared to (8) includes suggestions for overcoming barriers to digital tool use. Recommendations included attempts to reduce digital exclusion by providing training on digital tools in various modalities (e.g., videos, face-to-face) to increase research participant's/staff confidence in use and providing a physical location and/or digital tool in order for people to participate in a study. Further insights included issues around digital tool selection, participants proposed the need for a standardised process for selecting the most appropriate tool as well as published case studies of effective tools. Indeed, it has been previously reported that staff are often not sure of tool appropriateness for a particular task, research stage or stakeholder group and therefore a formalised selection process is required (1, 8). The need for collaboration with clinical staff and digital expertise was also highlighted in the context of developing digital tools in the future, case study 1 (Table S9, Supplementary materials) provided an example of a cost-effective bespoke tool.

Consistent with previous research, our study supports concerns around the security of data when using digital tools, particularly among older age research participants and invitations to participate via text/email (14, 21). It is important that research participants perceive invitations as authentic and legitimate otherwise this may negatively affect study uptake (8). In the present study, a participant reported conducting research to compare the recruitment rates of a pre-notification text to inform participants they would be shortly receiving a survey link compared with no text. However, no difference was found between the methods on participant uptake. This indicates such an approach may not be effective in improving the credibility of the subsequent message. It is critical that participants can trust recruitment invitations for clinical research, with the opportunity for scam messages being minimised, based on the way invitations are distributed.

For peer review only - http://bmjopen.bmj.com/site/about/guidelines.xhtml

This study highlights several considerations for research staff and organisations when utilising digital tools for research. Research projects should aim to be inclusive for people who may not have access to digital tools or the internet (e.g., providing a physical location/digital tool for people to complete the study) (1, 19). Organisations need to consider whether adequate training is provided for both staff and research participants to help address gaps in knowledge, demystify tools and increase confidence (1, 22). It is apparent that one size does not fit all, research staff should be willing to accommodate personal preferences of research participants when using digital tools (e.g., option to keep video camera off on video conferencing platforms) as well as consider offering a hybrid approach (both paper and digital tool options). In addition, staff should capitalise on digital expertise both locally in their organisations and nationally via funders and other relevant organisations.

Strengths of this study include the use of a broad range of stakeholders as participants and the use of mixed methods, enabling exploration of digital tool use from multiple perspectives. However, a limitation of this approach is the wide scope of the survey questions (i.e. covering all research stages) which does not allow deeper exploration into specific tools. It was apparent that many of the tools identified within the surveys were only referenced once by a single participant, suggesting a possible lack of consistency in the tools used within clinical research and the need for standardisation across each research stage. Alternatively, another explanation could be the small number of survey respondents which could make it difficult to generalise the results. Future research could elaborate on this work and investigate tools used in different geographical regions. A further limitation of this study is not exploring the views of participants/carers due to limited participant response. Given that our findings, alongside previous research, suggest that participants/carers are a key user of digital tools, future research should seek to understand their experiences, ensuring there are no barriers to accessing/using digital tools to prevent digital exclusion and promote diversity in participants. It is likely that participants were motivated to participate due to an underlying interest in digital tools, consequently some perspectives may not have been included in this study (staff, participants/carers who haven't used tools).

For peer review only - http://bmjopen.bmj.com/site/about/guidelines.xhtml

It is evident that future research should develop a number of resources to help researchers choose appropriate digital tools. Our research findings alongside previous studies have recognised requirements to publish case studies of effective digital tools (specific to research stage/stakeholders) as well as develop a standardised process of choosing a tool and/or programme for recommending appropriate tools (15). A target for the funders and organisations related to clinical research is to develop local and national networks to share and showcase expertise in digital tools. Further research avenues should include an evaluation of the effectiveness of tools (specific to the research stage) and an economic analysis to better understand the cost effectiveness of digital tools (particularly in relation to non-digital approaches) which would help to bolster the argument for use of digital tools in clinical research.

Contributors CC, TNA, CT and MTE obtained funding and conceptualised the research. ME and SC drafted initial online survey questions and qualitative interview questions; SC, CC, TNA, ME, MTE, CT, reviewed the draft survey/qualitative questions. SC, ME, CC, MEV were directly involved in activities relating to participant recruitment. SC and OR tidied the qualitative interview transcripts and analysed qualitative data. SC and MTE conducted data analysis for online survey results and drafted the manuscript, all authors reviewed the final manuscript.

Competing interests CC and MEV are employed by the funder (NIHR) of this project. The review is part of a wider project which CC helped to conceptualise. CC and MEV have revised and helped finalise the online survey and interview questions while also providing guidance about digital tools used in clinical research. CC and MEV were directly involved in activities relating to participant recruitment.

Funding This study was supported by the National Institute for Health and Care Research, Clinical Research Network West Midlands Improvement and Innovation Strategic Funding.

Data sharing statement All data relevant to the study are included in the article or uploaded as supplementary information.

Ethics Approval This study involves human participants and was approved by the University of Warwick's Biomedical and Scientific Research Ethics Sub-Committee (111/20-21). Participants gave informed consent to participate in the study before taking part

Acknowledgements The authors thank all study respondents taking part in the study. The authors also thank the steering group for their contributions as well as the participants who provided case study examples of effective digital tools.

References

1. Masoli JAH, Down K, Nestor G, Hudson S, O'Brien JT, Williamson JD, et al. A report from the NIHR UK working group on remote trial delivery for the COVID-19 pandemic and beyond. TRIALS. 2021;22(1).

For peer review only - http://bmjopen.bmj.com/site/about/guidelines.xhtml

2. Shamsuddin A, Sheikh A, Keers RN. Conducting Research Using Online Workshops During COVID-19: Lessons for and Beyond the Pandemic. International Journal of Qualitative Methods. 2021;20:16094069211043744.
3. Gesualdo F, Daverio M, Palazzani L, Dimitriou D, Diez-Domingo J, Fons-Martinez J, et al. Digital tools in the informed consent process: a systematic review. BMC Medical Ethics. 2021;22(1).
4. Darmawan I, Bakker C, Brockman TA, Patten CA, Eder M. The Role of Social Media in Enhancing Clinical Trial Recruitment: Scoping Review. J Med Internet Res. 2020;22(10):e22810.
5. Loucks TL, Tyson C, Dorr D, Garovic VD, Hill J, McSwain SD, et al. Clinical research during the COVID-19 pandemic: The role of virtual visits and digital approaches. Journal of Clinical and Translational Science. 2021;5(1):e102.
6. Chen ZM, Chen LR, Chen HF. The impact of COVID-19 on the clinical trial. PLOS ONE. 2021;16(5).
7. Department of Health & Social Care. The Future of Clinical Research Delivery: 2022 to 2025 implementation plan. 2022 30 June 2022.
8. Blatch-Jones A, Nuttall J, Bull A, Worswick L, Mullee M, Peveler R, et al. Using digital tools in the recruitment and retention in randomised controlled trials: Survey of UK Clinical Trial Units and a qualitative study. Trials. 2020;21(1).
9. Vorstenbosch E, Masoliver-Gallach R, Escuder-Romeva G. Measuring professional stigma towards patients with a forensic mental health status: protocol for a Delphi consensus study on the design of a questionnaire. BMJ Open. 2022;12(9):e061160.
10. Braun V, Clarke V. Using thematic analysis in psychology. Qualitative Research in Psychology. 2006;3(2):77-101.
11. Fisher LB, Tuchman S, Curreri AJ, Markgraf M, Nyer MB, Cassano P, et al. Transitioning From In-Person to Remote Clinical Research on Depression and Traumatic Brain Injury During the COVID-19 Pandemic: Study Modifications and Preliminary Feasibility From a Randomized Controlled Pilot Study. JMIR formative research. 2021;5(12):e28734.
12. Everhart RS, Dempster KW, Withers D, Mazzeo SE, Corona R, Thacker LR, 2nd, Schechter MS. Adaptations to the RVA Breathes clinical trial due to the COVID-19 pandemic. Contemporary clinical trials communications. 2021;24:100871.
13. Barakat S, Touyz S, Maloney D, Russell J, Hay P, Cunich M, et al. Supported online cognitive behavioural therapy for bulimia nervosa: a study protocol of a randomised controlled trial. Journal of Eating Disorders. 2021;9(1).
14. Rosa C, Marsch LA, Winstanley EL, Brunner M, Campbell ANC. Using digital technologies in clinical trials: Current and future applications. Contemporary Clinical Trials. 2021;100:106219.
15. Sydes MR, Barbachano Y, Bowman L, Denwood T, Farmer A, Garfield-Birkbeck S, et al. Realising the full potential of data-enabled trials in the UK: a call for action. BMJ Open. 2021;11(6):e043906.
16. Brennan CM, Nadella S, Zhao X, Dima RJ, Jordan-Martin N, Demestichas BR, et al. Oral famotidine versus placebo in non-hospitalised patients with COVID-19: a randomised, double-blind, data-intense, phase 2 clinical trial. Gut. 2022.
17. Alsem MW, Verhoef M, Braakman J, van Meeteren KM, Siebes RC, Jongmans MJ, et al. Parental empowerment in paediatric rehabilitation: Exploring the role of a digital tool to help parents prepare for consultation with a physician. Child: care, health and development. 2019;45(5):623-36.
18. Carpenter MJ, Gray KM, Wahlquist AE, Cropsey K, Saladin ME, Froeliger B, et al. A Pilot Randomized Clinical Trial of Remote Varenicline Sampling to Promote Treatment Engagement and Smoking Cessation. Nicotine & tobacco research : official journal of the Society for Research on Nicotine and Tobacco. 2021;23(6):983-91.
19. Inan OT, Tenaerts P, Prindiville SA, Reynolds HR, Dizon DS, Cooper-Arnold K, et al. Digitizing clinical trials. npj Digital Medicine. 2020;3(1):101.

For peer review only - http://bmjopen.bmj.com/site/about/guidelines.xhtml

20. Saeed SA, Masters RM. Disparities in Health Care and the Digital Divide. Current Psychiatry Reports. 2021;23(9):61.

21. Frampton GK, Shepherd J, Pickett K, Griffiths G, Wyatt JC. Digital tools for the recruitment and retention of participants in randomised controlled trials: a systematic map. Trials. 2020;21(1):478.

22. Mitsi G, Grinnell T, Giordano S, Goodin T, Sanjar S, Marble E, Pikalov A. Implementing Digital Technologies in Clinical Trials: Lessons Learned. Innov Clin Neurosci. 2022;19(4-6):65-9.

For peer review only

For peer review only - http://bmjopen.bmj.com/site/about/guidelines.xhtml

For peer review only

For peer review only - http://bmjopen.bmj.com/site/about/guidelines.xhtml

Supplementary Material

Table S1: Definition of digital tool by survey type

Research Staff	Research and Development
In this study a **digital tool** is defined as an alternative to paper-based methods that is IT based or an online platform that aids any aspect of the research study (stages of clinical research include but are not limited to; set up of research studies, participant recruitment, consent, participant retention, intervention delivery, data collection, data analysis, monitoring, patient reported outcomes).<br>Examples of digital tools might include but are not limited to:<br>• Electronic databases for participant screening<br>• Social media platforms for participant recruitment<br>• Communication within your research team (e.g., reporting adverse events via text message or email)<br>• Electronic consent<br>• Text messaging for participant retention<br>• Digital platforms for collecting survey data	In this study a **digital tool** is defined as an alternative to paper-based methods that is IT based or an online platform that aids any aspect of the research study set up, recruitment of participants and management.<br>Examples of digital tools might include but are not limited to:<br>• Electronic screening for participants at site level<br>• Remote monitoring via smart phone applications<br>• Local social media routes<br>• R&D department's website<br>• Text alerts

For peer review only

1

2 **Table S2:** Summary of Question area by survey type (Research Staff, R&D)

Survey Type	Question Themes
**Research Staff**	**Most effective digital tool**<br>· Description of tool<br>· Type of research digital tool used within (e.g., clinical trial)<br>· Stage of research project tool related to (e.g., recruitment)<br>· Project status<br>· Widely used/bespoke tool<br>· Costs associated with acquiring tool<br>· Training required to use tool<br>· End user of tool (drop down menu of pre-specified answers)<br>· Effectiveness of the digital tool relative to other digital tools (0 = not effective, 10 = very effective)<br>· How essential was digital tool to successfully implement the research (relative to alternative non-digital tools) (1 Highly non-essential to 5 Highly essential)<br>· Effectiveness of integration of digital tool with other health care or research systems (1 Not applicable to 5 highly effective)<br>· Level of technical literacy required to use/access tool (1 Very difficult to use/access to 4 Very easy to use/access)<br>· Main goal/s of tool (drop down menu of pre-specified answers)<br>Least effective digital tool<br>· Same as above except for Ineffectiveness of the digital tool relative to other digital tools 0 = no less effective than other tools, 10 = much less effective than other tools.
**R&D**	**Most effective digital tool example for set up**<br>· Description of tool<br>· How heard about digital tool<br>· Training (if yes number of days)<br>· Widely used/bespoke novel tool<br>· Cost information<br>· Perceived cost effectiveness of tool<br>Most effective digital tool example for recruitment<br>· Same as above<br>Most effective digital tool example for management

For peer review only

· Same as above

Table S3: Summary of interview question areas by participant type

Interview Type	Question Themes
Researchers and Related Staff	Definition of digital tools<br>How did COVID-19 affect role/use of digital tools<br>Case study about most effective tool<br>Case study about least effective tool<br>Any instances where tools could be used<br>Any barriers to digital tools<br>What would help researchers when deciding what tool to use/where to find this information
R&D Staff	Definition of digital tools<br>How did COVID-19 affect role/use of digital tools<br>Case study most effective tool for set up<br>Case study most effective tool for recruitment<br>Case study most effective tool for management<br>Any instances where tools could be used<br>Any barriers to digital tools<br>What would help researchers when deciding what tool to use/where to find this information

For peer review only

Table S4: Research staff and related survey participant characteristics

Number of Participants (N=41)	N (%)
County	
County of the West Midlands	26 (63.4)
Warwickshire	9 (22)
Staffordshire	4 (9.8)
Multiple counties in the CRN West Midlands	2 (4.9)

Employing organisation	
University	14 (34.1)
Clinical Trials Unit	13 (31.7)
NHS Trust	11 (26.8)
Other (please provide further details below)	3 (7.3)
Job role	
Chief Investigator	8 (19.5)
Other	8 (19.5)
Trial/Study Coordinator	8 (19.5)
Trial/Study Manager	8 (19.5)
Research Manager	3 (7.3)
Principal Investigator	2 (4.9)
Research Nurse	2 (4.9)
Trial/Study Data Manager	2 (4.9)

For peer review only

Table S5 Most effective and least effective digital tools results

Question Type	Answer Options	Most Effective N(%)	Least Effective N (%)
Type of research project	Clinical Trial	8 (38.1)	9 (42.9)
	Clinical Research	6 (28.6)	6 (28.6)
	Health Research	5 (23.8)	4 (19)
	Other	1 (4.8)	1 (4.8)
	Social Care	1 (4.8)	1 (4.8)
	Total number of responses	21	21
Stage of research	Research study set up	2 (9.5)	4 (19)
	Participant recruitment	4 (19)	8 (38.1)
	Data collection	11 (52.4)	6 (28.6)
	Intervention delivery	2 (9.5)	1 (4.8)
	Quality Assurance	n/a	1 (4.8)
	Outcome measures	1 (4.8)	n/a

For peer review only - http://bmjopen.bmj.com/site/about/guidelines.xhtml

	Other	1 (4.8)	1 (4.8)
	Total number of responses	21	21
Project status	Completed	4 (19)	4 (19)
	Ongoing	17 (81)	17 (81)
	Total number of responses	21	21
Widely used vs Bespoke digital tool	Novel/bespoke to my organisation	4 (19)	3 (14.3)
	Novel/bespoke to my project team	5 (23.8)	1 (4.8)
	Used widely	12 (57.1)	17 (81)
	Total number of responses	21	21
Costs associated with acquiring digital tool	A one-off up-front fee	1 (4.8)	1 (4.8)
	A one-off up-front fee, periodic recurring fee	2 (9.5)	1 (4.8)
	I'm not sure	4 (19)	8 (38.1)
	No fee (e.g., open source)	8 (38.1)	7 (33.3)
	Other	3 (14.3)	3 (14.3)
	Periodic recurring fee	3 (14.3)	1 (4.8)
	Total number of responses	21	21
Training required in order to use tool	No	11 (52.4)	12 (57.1)
	Yes	10 (47.6)	9 (42.9)
	Total number of responses	21	21
How essential was this tool to successfully implement the research	Somewhat non-essential (2)	1 (4.8)	4 (19)
	Somewhat essential (3)	8 (38.1)	9 (42.9)
	Highly essential (4)	12 (57.1)	8 (38.1)
	Total number of responses	21	21
Integration with other healthcare or research systems	Not applicable (1)	3 (14.3)	2 (9.5)
	Somewhat ineffective (3)	3 (14.3)	6 (28.6)
	Somewhat effective (4)	8 (38.1)	10 (47.6)
	Highly effective (5)	7 (33.3)	3 (14.3)
	Total number of responses	21	21

For peer review only

Level of technical literacy required	Somewhat difficult to use/access (2)	1 (4.8)	7 (33.3)
	Somewhat easy to use/access (3)	10 (47.6)	10 (47.6)
	Very easy to use/access (4)	10 (47.6)	4 (19)
	Total number of responses	21	21

Table S6: Rating of effectiveness of most effective tool (0 no more effective to 10 much more effective)

Answer Options		Rating of most effective tool compared to other tools
	0	n/a
	1	n/a
	2	n/a
	3	n/a
	4	n/a
	5	1 (4.8%)
	6	1 (4.8%)
	7	4 (19%)
	8	7 (33.3%)
	9	5 (23.8%)
	10	3 (14.3%)
Total number of responses		21

Table S7: Rating of ineffectiveness of least effective tool (0 = no less effective than other tools, 10 = much less effective than other tool)

For peer review only

Answer Options	Rating of least effective tool compared to other tools
0	1 (5%)
1	1 (5%)
2	2 (10%)
3	2 (10%)
4	3 (15%)
5	7 (35%)
6	1 (5%)
7	2 (10%)
8	1 (5%)
9	n/a
10	n/a
Total number of responses	20

Table S8: R&D Survey Participant Characteristics

Number of Participants (N=25)	N(%)
Experience Using Digital Tools	
Participants who used digital tools	22(88)
Participants who have not used digital tools	3(12)
County	
County of the West Midlands	10(40)
Warwickshire	5(20)
Multiple counties in CRN West Midlands	3(12)

For peer review only

Staffordshire	3(12)
Worcestershire	2(8)
Herefordshire	1(4)
Shropshire	1(4)
Employing Organisation	
NHS	25(100%)
Job Role	
R&D Manager/Head of R&D	13(52)
Lead Research Nurse	3(12)
Other (please provide further details below)	3(12)
R&D Facilitator (governance, study set-up)	3(12)
Data Manager	1(4)
Research Delivery staff	1(4)

1

For peer review only

BMJ Open- Draft 1 (SC)
May 2023

Table S9: Most effective tool case studies for management and recruitment

**Case study 1: Digital Tool for Management**	**Example Statements**
Two participants referred to a bespoke management tool as their most effective digital tool. The tool was designed and developed by R&D staff supported by a digital team embedded in an R&D department in collaboration with clinical research staff (primarily nurses). Built in excel, the tool is used to manage patients through a study protocol. Below, one participant describes the tool.	*"It meets multiple needs, so we build them individually off the back of each protocol…so when you put the patients recruited date it then predicts all their follow up visits for you in keeping with the protocol. It then has a traffic light system which would tell you when they're due to come up so the teams can see it and have oversight quite quickly of all the participants where they are, what happens at each individual stage. You then hover your mouse over visit 2 and a little info box will come up and tell you a brief synopsis of what needs to be done at that visit. So going quickly, either give you what needs to be done, you can click on it, and it takes you through to the worksheets that we create off the back of it."*
One participant explained the primary reason for developing the tool was to standardise study management.	*"It came around a sort of a want to offer standardised high-quality research and oversight... I've worked in lots of different research areas, and they've always got slightly different way of working. Some of the nurses and the clinical teams have sort of had to go at building their own Excel spreadsheets. Some of the sponsors and the studies provided them for us and then some used paper diaries. So, but when you look at it, none of them were quite really fulfilling everyone's needs, there were sort of doing a bit here and a bit there and I wanted to standardize it across the whole of R&D because it wasn't quite equitable for our patients and our staff. Some were getting access to. So that's why we standardized and went we're gonna build these and offer them out across the board."*
Prior to development of this bespoke management tool, it appears that studies were managed via paper means and/or nurses had attempted to build their own databases to manage studies.	*"Nurses that trying to build databases, you know, it's not our skill set, but some can do sort of just a small amount with them and then others were very much using paper which is not the way to do it."*
Both participants deemed the bespoke management tool to be effective for a number of different reasons including helping staff keep patients to the protocol time frames. One participant explained that the tool can be tweaked to suit needs of the users (i.e., nurses) and new features can be added. They also explained that it has many benefits due to being developed in excel, including tool template can be sent to teams located at different sites and used without the R&D staff visiting that particular site as well as being user friendly.	*"In terms of what it gains and what it offers for clinical deliveries…if you use [tool name] your patient should stay within [the] protocol visit window…and you can plan a lot easier, and again should someone go off…someone can come in and pick it up quite easily and you can ensure the patient is safe and kept up to date."*<br><br>*"One of the benefits of it is we've built it in Excel. It doesn't look like excel when you use it. Everyone's got excel within the trust, and there's no additional cost apart from the hours to build it, we weren't having to purchase another system. So that was why we chose that setup to build it. And then we're working with other sites and helping them have access. We can send them a template, we don't need to go there…they upload the template, then they can use it themselves. So that's why we built it that way just for that reason."*<br>*"I think we're on version seven or eight now, so they're really user friendly and they're really locked down so you can't break them. I've had some nurses do some really weird things and they've broken [it] but generally as times gone by, we've ironed out all the things. Before if a nurse dragged a formula*

For peer review only

	away and deletes it, it would affect everything else…but in the department we've [become] more savvy at locking things down. Generally, …you should be able to click and use it."
A further benefit highlighted by participants is the fact that the tool was developed in collaboration with clinical research staff and has incorporated their ideas into the tool such as automatically generating reports opposed to looking through paper documents.	*"It's made by the people delivering research…I think together we've developed the service over the years and the tracker itself and that's what makes it better than an out-of-the-box bit of kit from it or third-party developer…one of the things we built in which was one of the ideas from a nurse, she had nearly a 1000 patients on a study but she just wanted to see who she needed to see this month, and rather than scrolling down the 1000 patients with a pen seeing "Who do I need to see?" She clicks the month button, and it will automatically generate your report in PDF and just say these are your patients for the month…I think that's really where the track of kind of stands out. It's just that bit of being able to organize and manage yourself and your team a lot more effectively and actually you don't want to spend time trawling through bits of paper, paper calendars and diaries."*

**Case Study 2: Digital Tool for Recruitment**	**Example Statements [Participant Number]**
One participant described digital methods used in a clinical trial to recruit participants who meet the inclusion criteria of a study. Using data from the CPRD, researchers are able to know how many patients within each GP surgery meet an inclusion criteria for a study. Advantages of this include reduction in time and money spent on recruitment, as well as increases the likelihood of recruiting relevant participants. It is also beneficial for the patient, as the GP surgery may have relevant information on file that research studies can use (e.g., blood pressure). This is in turn also reduces the workload for the research team.	*"Practices sign up to CPRD [Clinical Practice Research Datalink] and they've got around 20% of practices across England. So CPRD are able to extract their coded medical data. So, any information that when you go to the GP and they say you've got diabetes, they put what they call a medical code in your patient records. So, you've got diabetes… CPRD able to extract that medical code...it's just that coded that this patient has diabetes, their blood pressure was this, they're on these drugs. They're able to extract that from practice records. So CPRD and [employing university] have spotted that actually there's a potential for using this in an interventional trial. So, it means there is an ability to extract that medical info, we can first of all pre-screen remotely the entire of CPRD's population, so over 13 million records we're able to pre-screen against our trial inclusion and exclusion criteria. So, we're then able to actually instead of the old-fashioned research way, which is you ask practices that do they want to take part in research? They say yes. Sounds interesting. You set them up, you spend time, money, setting them up, and actually they've got one patient…they haven't got the patients you need. Whereas with this ability of CPRD's, we're able to know that practice has this amount of patients…we're going to those practices that are large and potentially successful in actually taking part in research so that means obviously it reduces the time that we spend, the money that we spend and hopefully increases the success of the trial…and also obviously reduces practice workload... every so often a patient would go into the practice, have their blood pressure taken anyway for us then to ask that patient to come in for an extra research visit when actually we can just pull that data from the medical record, it seems counterintuitive, so making it easier one for practices they don't have to arrange for patients to come in and two for patients, it means they can absolutely take part in this trial without actually having to go into their GP practice."*

The participant explained that they also used e-consent in the trial when recruiting participant. Consent forms were sent to patients from the GP practises via text message or email. The participant explains the process below.

"In response to COVID and the reduction in face-to-face GP visits we obviously needed to find a work around practices to be able to recruit patients to the trial. So, we actually developed a fully remote consent system using REDCap…we used it to send e-consent forms out to patients. So, the GP practice puts in some contact details of the patient and that then automatically sends a text message or e-mail to the patient containing a link to their consent form in this sort of electronic survey form. Patients then click on that link in that text message or e-mail it brings up their e-consent form. It provides them with the patient information sheet that they need to complete. Then they then go down and click yes, yes or no to each of the consent criteria. They then fill in their details. They're able to electronically sign that with that signature being saved so it matches that, that sort of the NHS criteria for e-consent system we then collect further information, so their e-mail and telephone number.

The participant explains below the benefits of participants, GP and research team all receiving a copy of the e-consent form

"and then once they've completed that it automatically notifies the GP practice that they've completed…the GP practice are then able to sign their section of the consent form and then it also automatically sends a copy of the consent form to the patient and to the practise. So, it automatically means that thinking about that good clinical practise the patients gets it, the practise gets it and us as a study team have that as a central repository so it makes it a lot easier for patient to consent… GP's obviously like that nature of being able to send the consent form out and then just being notified to say it's complete as well".

Other benefits include the ability to send out follow up questions in different languages.

"We're able to use REDCap to automatically send out our follow up questionnaires. So, every six months we send quality of life questionnaires that again, patients are able to do on their phone, they're able to do in different languages. And they're able to then that would so complete that automatically comes back us and saves in the REDCap database… it's able to be used by patients and we've always had good response and patients have found it easy to complete and we get really good sort of follow up questionnaire response rate. I think we've got very few that haven't completed their follow up questionnaires"

For peer review only

Supplementary Information 2a - Online Survey: Researchers and Related Staff

Start of Block: information sheet

Information sheet **PROJECT TITLE**: **NIHR Experiences of using Digital Tools in Clinical, Health and Social Care Research**

NAMES OF RESEARCHERS: Sophie Clohessy1, Prof Theo Arvanitis1, Dr Carla Toro1, Dr Mark Elliott1 Institute of Digital Health Care, WMG, University of Warwick)

NAME OF COLLABORATORS: Mark Evans2,3 Carly Craddock2,3 (2. The Royal Wolverhampton NHS Trust, Wolverhampton, UK, 3) National Institute for Health Research Clinical Research Network West Midlands, Birmingham, UK).

This sheet seeks to provide information, and advice, with respect to an individual's participation in support of the specified research project:

1. The study aims to investigate how online and digital tools/methods are being used for clinical, health and social care research. We are particularly interested to determine which approaches work well and which don't and use these findings to share best practice with the clinical, health and social care research community.

2. This study is funded by the National Institute of Health Research Clinical Research Network West Midlands (CRN West Midlands). The funding for this project has been awarded to Sophie Clohessy, Prof Theo Arvanitis, Dr Carla Toro, Dr Mark Elliott WMG, University of Warwick. The project will be led by The University of Warwick.

3. Participation in this research is totally voluntary, and assurances are given to the effect that no negative consequences will arise from refusal to participate in the research project.

4. Your consent for your data to be used in this questionnaire will be gained by your ticking the consent question at the end of this page, so by ticking this box you agree that your submitted data can be used in the aforementioned study.

5. Your data will be collected anonymously, therefore due to the anonymity of the data at source, once completed it is not possible for participants to withdraw their data from the study. However, you will be given the option to provide your name and contact details if you are happy

For peer review only - http://bmjopen.bmj.com/site/about/guidelines.xhtml

for us to contact you for further information based on your responses. Those who provide these details have the option to withdraw from the study up to 7 days after submission, by contacting Sophie Clohessy, s.clohessy@warwick.ac.uk.

6. Each individual is advised to fully consider, with others if necessary and prior to participation, any disadvantages, side effects, risks and/or discomforts that may arise from participation in this research.

7. Unless specifically agreed otherwise, all information will be held as confidential and will not be distributed outside of the research team.

8. Your data may be used as a source for future research, including research work for publication.

This research has been favourably reviewed by the University's Biomedical and Scientific Research Ethics Committee, Approval Reference: BSREC 111/20-21 dated: 17/01/2022. Dissatisfaction with the conduct of this research may be referred to the person below, who is a senior University of Warwick official entirely independent of this study: Head of Research Governance, Research & Impact Services, University House, University of Warwick, Coventry, CV4 8UW; Tel: 024 76 75733; Email: researchgovernance@warwick.ac.uk

consentform **Before you continue please understand that you are confirming your agreement with the following:**

- ○ I give my consent to my data submitted within this questionnaire being used for the purposes stated above including being happy for my data to be used in future research (1)
- ○ I do not consent, and I do not wish to take part (2)

Skip To: End of Survey If Before you continue please understand that you are confirming your agreement with the following: = I do not consent, and I do not wish to take part

Page Break

For peer review only - http://bmjopen.bmj.com/site/about/guidelines.xhtml

End of Block: information sheet

Start of Block: Work in West Midlands

Age **Are you aged 18 or over?**

- Yes (1)
- No (2)

Display This Question:

If Are you aged 18 or over? = No

End of survey **Thank you for your interest in our survey. Unfortunately we are only able to accept responses from people who are aged 18 or over.**

Please click on the next page to end the survey.

Skip To: End of Survey If Thank you for your interest in our survey. Unfortunately we are only able to accept responses fro... Is Displayed

Page Break

For peer review only - http://bmjopen.bmj.com/site/about/guidelines.xhtml

County **Which county in the Clinical Research Network West Midlands region is your employing organisation based within?**

▼ Shropshire (1) ... I don't work in any of these counties (7)

Display This Question:

If Which county in the Clinical Research Network West Midlands region is your employing organisation... = I don't work in any of these counties

End of survey **Thank you for your interest in our survey. Unfortunately we are only interested in responses from people who work within the Clinical Research Network West Midlands region.**

Please click on the next page to end the survey.

Skip To: End of Survey If Thank you for your interest in our survey. Unfortunately we are only interested in responses from... Is Displayed

End of Block: Work in West Midlands

Start of Block: Current use of digital tools

Use of tools
Please think about the clinical, health or social care research that you are currently working on or have worked on in the last two years. We are interested to learn whether you have used any **digital tools** during any stage of your research studies.

In this study a **digital tool** is defined as an alternative to paper based methods that is IT based or an online platform that aids any aspect of the research study (stages of clinical research include but are not limited to; set up of research studies, participant recruitment, consent, participant retention, intervention delivery, data collection, data analysis, monitoring, patient reported outcomes).

Examples of digital tools might include but are not limited to: Electronic databases for participant screening Social media platforms for participant recruitment Communication within your research team (e.g., reporting adverse events via text message or email) Electronic consent Text messaging for participant retention Digital platforms for collecting survey data

For peer review only - http://bmjopen.bmj.com/site/about/guidelines.xhtml

Thinking about the clinical, health or social care research studies you are currently working on or have worked over the past two years, have you used digital tools to assist in the operation, management or coordination of these studies?

- ◯ Yes (1)
- ◯ No (2)
- ◯ I have delegated digital tool use to colleagues (3)

Display This Question:

If Please think about the clinical, health or social care research that you are currently working on... = I have delegated digital tool use to colleagues

Q238 **Thank you for your interest in our survey. Please forward the link of this survey to a relevant team member.**

Skip To: End of Survey If Thank you for your interest in our survey. Please forward the link of this survey to a relevant t... Is Displayed

---

Display This Question:

If Please think about the clinical, health or social care research that you are currently working on... = No

X→

For peer review only

If not used digital

What barriers have you encountered that have prevented you using digital tools in your clinical, health or social care research? Please select all answer/s that apply.

- ☐ Lack of budget (1)
- ☐ Stick to what worked in the past (2)
- ☐ I wasn't aware of digital tools I could use (3)
- ☐ I would need training to use digital tools (4)
- ☐ I was concerned this would bias participant recruitment (5)
- ☐ Other (6) ____

Display This Question:

If Please think about the clinical, health or social care research that you are currently working on... = No

Intentions

Please rate the following statement on a 1 - 5 scale (1 "strongly disagree") and (5 "strongly agree")

"I intend to use digital tools in future clinical, health or social care research studies"

For peer review only

- ○ 1 Strongly disagree (1)
- ○ 2 (2)
- ○ 3 (3)
- ○ 4 (4)
- ○ 5 Strongly agree (5)

Page Break

For peer review only

For peer review only - http://bmjopen.bmj.com/site/about/guidelines.xhtml

Display This Question:

If Please think about the clinical, health or social care research that you are currently working on... = No

Opt in non digital **By taking part in this survey, you also have the opportunity to take part in a qualitative one to one interview (most likely conducted remotely and at a time convenient for you). We anticipate the interview will take approximately between 30 and 60 minutes.**

During this interview, we will explore reasons for not using digital tools in your clinical, health or social care research in further depth and your views towards these tools.

If you are interested in taking part, please provide your name and email address below and we will be in touch shortly to provide further details.

- ◯ Yes I am interested in providing my contact details (1)
- ◯ No, I opt out of taking part in a qualitative interview (2)

Display This Question:

If By taking part in this survey, you also have the opportunity to take part in a qualitative one to... = No, I opt out of taking part in a qualitative interview

End of survey **Please click on the next page to complete the survey.**

Skip To: End of Survey If Please click on the next page to complete the survey. Is Displayed

---

Display This Question:

If By taking part in this survey, you also have the opportunity to take part in a qualitative one to... = Yes I am interested in providing my contact details

Full name **Full Name**

____

Display This Question:

If By taking part in this survey, you also have the opportunity to take part in a qualitative one to... = Yes I am interested in providing my contact details

For peer review only - http://bmjopen.bmj.com/site/about/guidelines.xhtml

Email address **Email Address**

____

Display This Question:

If By taking part in this survey, you also have the opportunity to take part in a qualitative one to... = Yes I am interested in providing my contact details

Job title **Job Title**

____

Display This Question:

If By taking part in this survey, you also have the opportunity to take part in a qualitative one to... = Yes I am interested in providing my contact details

Organisation **Which organisation do you work for?**

____

Display This Question:

If By taking part in this survey, you also have the opportunity to take part in a qualitative one to... = Yes I am interested in providing my contact details

complete survey **Please click on the next page to complete the survey.**

Skip To: End of Survey If Please click on the next page to complete the survey. Is Displayed

End of Block: Current use of digital tools

Start of Block: Introduction

Instructions **On the next page we will ask you some questions about you.**

End of Block: Introduction

Start of Block: Demographics

X→

For peer review only - http://bmjopen.bmj.com/site/about/guidelines.xhtml

General role **What is/was your role in the most recent clinical, health or social care research you were involved with?**

- ◯ Chief Investigator (3)
- ◯ Clinical Researcher (1)
- ◯ Data Analyst (9)
- ◯ Principal Investigator (2)
- ◯ Research Manager (7)
- ◯ Research Nurse (8)
- ◯ Trial/Study Coordinator (4)
- ◯ Trial/Study Data Manager (6)
- ◯ Trial/Study Manager (5)
- ◯ Other (please provide further details below) (1)

____

Organisation **What organisation do you work for?**

- ◯ Clinical Trials Unit (2)
- ◯ NHS Trust (4)
- ◯ Primary Care (3)
- ◯ University (1)
- ◯ Other (please provide further details below) (5)

____

For peer review only - http://bmjopen.bmj.com/site/about/guidelines.xhtml

Place of work **Please provide the specific place of work (optional)**

____

End of Block: Demographics

Start of Block: All examples

Examples **Please provide a list of digital tools you are currently using or have used in your clinical, health or social care research studies over the last two years (including those still running). You can provide as many examples as you like.**

Examples **List of digital tools used in the last two years**

____

____

____

____

____

Page Break

For peer review only

Instructions **We will now ask you some more in depth questions about two of the digital tools you provided.**

Please choose one digital tool which you believe worked MOST effectively and one you believe worked LEAST effectively, in terms of operating/managing/coordinating the research study.

Please consider your examples based on any clinical/health/social research projects you have worked on in the last 2 years (including those still running)

We will ask you a block of questions about each example. On the next page, we will ask you about your first example.

End of Block: All examples

Start of Block: Least effective tool

Intro We will now ask you a block of questions about a digital tool you have used that has worked LEAST EFFECTIVELY in terms of operating, managing or coordinating a clinical/health/social research study.

Please consider your answer based on any clinical/health/social research projects you have worked on in the last 2 years (including those still running)

Page Break

For peer review only

For peer review only - http://bmjopen.bmj.com/site/about/guidelines.xhtml

Least effective eg

Please state which digital tool you have used that worked LEAST EFFECTIVELY in terms of operating/managing/coordinating a clinical/health/social research study?

Please provide a very brief explanation of the tool/method.

(You can additionally provide a website link to a publication or trial website.)

____

____

____

____

____

Page Break

For peer review only

For peer review only - http://bmjopen.bmj.com/site/about/guidelines.xhtml

Least eff stage
This an example of a digital tool relating to...
(please select your answer using the drop down box)

▼ Data analysis (1) ... Other (10)

Least eff effective **Relative to your experience of other digital tools used in clinical research, how ineffective do you rate this tool?**

0 = no less effective than other tools, 10 = much less effective than other tools.

	0	1	2	3	4	5	6	7	8	9	10
9 ()											

Least eff details **In what way is/was the tool/platform ineffective?**

____

____

____

____

____

Least advantages **Based on your experience, please list any advantages that the use of this digital tool has brought to your clinical, health or social care research (If applicable).**

____

____

____

For peer review only - http://bmjopen.bmj.com/site/about/guidelines.xhtml

Least disadvantages
Based on your experience, please list any <u>disadvantages</u> that the use of this digital tool has brought to your clinical, health or social care research (If applicable).

____

____

____

____

____

Least eff status **You have provided a digital tool from a project you are currently working on or previously worked on. What is the status of this project?**

- ○ Setting up (1)
- ○ Ongoing (2)
- ○ Completed (3)

Study managing **What is/was the study speciality area?** (please select your answer using the drop down box)

▼ Ageing (1) ... Other (31)

For peer review only - http://bmjopen.bmj.com/site/about/guidelines.xhtml
For peer review only

Least eff study type
This digital tool was used in ...

- ◯ A Clinical Trial (2)
- ◯ Clinical Research (1)
- ◯ Health Research (3)
- ◯ Social Care Research (4)
- ◯ Other (5) ____

Least eff user **Who is/was the end user of this digital tool? (Please tick all that apply)**

- ▢ Carer/family member of research participant (6)
- ▢ Front line health and care staff (1)
- ▢ Principal Investigators (3)
- ▢ Research delivery workforce (2)
- ▢ Research participants (4)
- ▢ Other/Multiple users (Further information can be added in the text box) (5)

____

Page Break

For peer review only

For peer review only - http://bmjopen.bmj.com/site/about/guidelines.xhtml

Least eff goal **What is/was the main goal of this digital tool? (Please tick all that apply)**

- ▢ Deal with Covid related restrictions (3)
- ▢ Facilitate good data management / Information governance (6)
- ▢ Increase patient accessibility (2)
- ▢ Improve data handling for analysis/modelling (5)
- ▢ Reduce time/Increase efficiency (1)
- ▢ Other (7) ____

Previous method repl **What previous (i.e., non-digital/paper-based) method did this digital tool replace?**

____

Least novelty **Is this digital tool novel/bespoke to your project team or used widely?**

- ○ Novel/bespoke to my project team (1)
- ○ Novel/bespoke to my organisation (2)
- ○ Used widely (3)

Page Break

For peer review only - http://bmjopen.bmj.com/site/about/guidelines.xhtml

X→

least eff implement **How essential was this tool to successfully implement the research (relative to alternative non-digital tools)?**

○ Highly non-essential 1 (1)

○ Somewhat non-essential 2 (2)

○ Somewhat essential 3 (3)

○ Highly essential 4 (4)

Page Break

For peer review only

For peer review only - http://bmjopen.bmj.com/site/about/guidelines.xhtml

Least eff integ **How effective was/is this tool at integrating with other healthcare or research systems used during your research?**

- Not applicable 1 (1)
- Highly ineffective 2 (2)
- Somewhat ineffective 3 (3)
- Somewhat effective 4 (4)
- Highly effective 5 (5)

Page Break

For peer review only

For peer review only - http://bmjopen.bmj.com/site/about/guidelines.xhtml

Least eff tech lit **In your opinion, what level of technical literacy is/was required to use or access this digital tool?**

- Very difficult to use/access 1 (1)
- Somewhat difficult to use/access 2 (2)
- Somewhat easy to use/access 3 (3)
- Very easy to use/access 4 (4)

Page Break

For peer review only

Least eff training **Did you require training in order to use this digital tool?**

○ Yes (1)

○ No (2)

Page Break

For peer review only

Least eff costs **What costs were associated with acquiring this tool? (Please tick all that apply)**

- ☐ A one-off up-front fee (e.g. outright purchase) (1)
- ☐ Periodic recurring fee (e.g. monthly/annual license/maintenance fee) (2)
- ☐ No fee (e.g. open source) (3)
- ☐ Other (4) ____
- ☐ I'm not sure (5)

Least eff comments **Please provide any further comments or information in the text box below about your digital tool example (optional)**

____
____
____
____
____

End of Block: Least effective tool

Start of Block: Most effective tool

Intro We will now ask you a block of questions about a digital tool you have used that worked MOST EFFECTIVELY in terms of operating, managing or coordinating a clinical/health/social research study.

Please consider your answer based on any clinical/health/social research projects you have worked on in the last 2 years (including those still running)

For peer review only

For peer review only - http://bmjopen.bmj.com/site/about/guidelines.xhtml

Page Break

For peer review only

For peer review only - http://bmjopen.bmj.com/site/about/guidelines.xhtml

Most effect e.g

Please state which digital tool you have used worked MOST EFFECTIVELY in terms of operating/managing/coordinating clinical/health/social research?

Please provide a very brief explanation of the tool/method.

(You can additionally provide a website link to a publication or trial website.)

Page Break

For peer review only

Most eff stage

This an example of a digital tool relating to...

(please select your answer using the drop down box)

▼ Data analysis (1) ... Other (10)

Most eff effective **Relative to your experience of other digital tools used in clinical research, how effective do you rate this tool?**

0 = no more effective than other tools, 10 = much more effective than other tools.

	0	1	2	3	4	5	6	7	8	9	10
9 ()											

Most eff details **In what way is/was the tool/platform effective?**

Most eff advantages **Based on your experience, please list any <u>advantages</u> that the use of this digital tool has brought to your clinical, health or social care research (If applicable).**

For peer review only - http://bmjopen.bmj.com/site/about/guidelines.xhtml

Most eff disadvantag
Based on your experience, please list any disadvantages that the use of this digital tool has brought to your clinical, health or social care research (If applicable)

____

____

____

____

____

Most eff status **You have provided a digital tool from a project you have worked/currently working on. What is the status of this project?**

- Setting up (1)
- Ongoing (2)
- Completed (3)

Most eff speciality **What is/was the study speciality area?**
(please select your answer using the drop down box)

▼ Ageing (1) ... Other (31)

For peer review only

For peer review only - http://bmjopen.bmj.com/site/about/guidelines.xhtml

Most eff
This digital tool was used in ...

- ○ A Clinical Trial (2)
- ○ Clinical Research (1)
- ○ Health Research (3)
- ○ Social Care (4)
- ○ Other (5) ____

Most eff user **Who is/was the end user of this digital tool? (Please tick all that apply)**

- ▢ Carer/family member of research participant (6)
- ▢ Front line health and care staff (1)
- ▢ Principal Investigators (3)
- ▢ Research delivery workforce (2)
- ▢ Research participants (4)
- ▢ Other/Multiple users (Further information can be added in the text box) (5)

____

Page Break

For peer review only - http://bmjopen.bmj.com/site/about/guidelines.xhtml

Most eff end goal **What is/was the main goal of this digital tool? (Please tick all that apply)**

- ☐ Deal with Covid related restrictions (3)
- ☐ Facilitate good data management / Information governance (6)
- ☐ Increase patient accessibility (2)
- ☐ Improve data handling for analysis/modelling (5)
- ☐ Reduce time/Increase efficiency (1)
- ☐ Other (7) ____

most eff previous **What previous (i.e., non-digital/paper-based) method did this digital tool replace?**

____

Most eff novelty **Is this digital tool novel/bespoke to your project team or used widely?**

- ○ Novel/bespoke to my project team (1)
- ○ Novel/bespoke to my organisation (2)
- ○ Used widely (3)

Page Break

For peer review only - http://bmjopen.bmj.com/site/about/guidelines.xhtml

most eff implement **How essential was this tool to successfully implement the research (relative to alternative non-digital tools)?**

- Highly non-essential 1 (1)
- Somewhat non-essential 2 (2)
- Somewhat essential 3 (3)
- Highly essential 4 (4)

Page Break

For peer review only - http://bmjopen.bmj.com/site/about/guidelines.xhtml

Most eff integration **How effective was/is this tool at integrating with other healthcare or research systems used during your research?**

- ◯ Not applicable 1 (1)
- ◯ Highly ineffective 2 (2)
- ◯ Somewhat ineffective 3 (3)
- ◯ Somewhat effective 4 (4)
- ◯ Highly effective 5 (5)

Page Break

For peer review only

Most eff tech lit **In your opinion, what level of technical literacy is/was required to use or access this digital tool?**

- Very difficult to use/access 1 (1)
- Somewhat difficult to use/access 2 (2)
- Somewhat easy to use/access 3 (3)
- Very easy to use/access 4 (4)

Page Break

For peer review only

Most eff training **Did you require training in order to use this digital tool?**

- ◯ Yes (1)
- ◯ No (2)

Page Break

For peer review only

For peer review only - http://bmjopen.bmj.com/site/about/guidelines.xhtml

Most eff costs **What costs were associated with acquiring this tool? (Please tick all that apply)**

- ▢ A one-off up-front fee (e.g. outright purchase) (1)
- ▢ Periodic recurring fee (e.g. monthly/annual license/maintenance fee) (2)
- ▢ No fee (e.g. open source) (3)
- ▢ Other (4) ____
- ▢ I'm not sure (5)

Most eff comments **Please provide any further comments or information in the text box below about your digital tool example (optional)**

____

____

____

____

____

End of Block: Most effective tool

Start of Block: Technology Anxiety

Tech anxiety
Technology Anxiety

Think about the digital tool example you provided. Please rate the following statements

For peer review only - http://bmjopen.bmj.com/site/about/guidelines.xhtml

on a "Strongly Disagree" to "Strongly Agree" scale.

	Strongly Disagree (1)	Very Much Disagree (2)	Disagree (4)	Not Sure (5)	Agree (6)	Very Much Agree (7)	Strongly Agree (8)
I like to keep up with new technology (11)	○	○	○	○	○	○	○
I am open to change my work habits if necessary (20)	○	○	○	○	○	○	○
I am likely to become technology-oriented due to the nature of my field (21)	○	○	○	○	○	○	○
If I were given enough time and sufficient resources to absorb change, I am likely to accept it (22)	○	○	○	○	○	○	○
Overall, I am not that anxious about using technology (23)	○	○	○	○	○	○	○

End of Block: Technology Anxiety

For peer review only

For peer review only - http://bmjopen.bmj.com/site/about/guidelines.xhtml

Start of Block: Opt in interviews

Qualitative survey **By taking part in this survey, you also have the opportunity to take part in a qualitative one to one interview (most likely conducted remotely and at a time convenient for you). We anticipate the interview will take between 30-60 minutes.**

During this interview, we will explore your experiences about using digital tools in clinical research in further depth. We will ask you questions about the information you provided in this survey.

This study is part of a wider project to learn from people who have used digital tools within their research, learn about the benefits and drawbacks of these methods and inform other researchers about the digital tools available to them.

If you are interested in taking part, please provide your name and email address below and we will be in touch shortly to provide further details.

○ Yes I am interested in providing my contact details and I consent to the answers on this survey being discussed during the interview (1)

○ No, I opt out of taking part in a qualitative interview (2)

Display This Question:

If By taking part in this survey, you also have the opportunity to take part in a qualitative one to... = Yes I am interested in providing my contact details and I consent to the answers on this survey being discussed during the interview

Full name **Full Name**

____

Display This Question:

If By taking part in this survey, you also have the opportunity to take part in a qualitative one to... = Yes I am interested in providing my contact details and I consent to the answers on this survey being discussed during the interview

Email address **Email Address**

____

For peer review only - http://bmjopen.bmj.com/site/about/guidelines.xhtml

Display This Question:

If By taking part in this survey, you also have the opportunity to take part in a qualitative one to... = Yes I am interested in providing my contact details and I consent to the answers on this survey being discussed during the interview

Job role **Job Title**

____

Display This Question:

If By taking part in this survey, you also have the opportunity to take part in a qualitative one to... = Yes I am interested in providing my contact details and I consent to the answers on this survey being discussed during the interview

Name of organisation **Which organisation do you work for?**

____

End of Block: Opt in interviews

Start of Block: Free text box

Further Information **Please use this text box to provide any further information that you think may be relevant about your experience of using digital tools in your clinical research.**

____

____

____

____

____

For peer review only

comments **Please leave any comments about the study below. In particular, please tell us if there was anything unclear or confusing about the study or any questions that you think we should include next time.**

____

____

____

____

____

End of Block: Free text box

Start of Block: Debriefing sheet

Debriefing sheet
Debriefing sheet

Study title: Digitising Clinical Research

Thank you for taking part in the questionnaire-based study. Researchers Sophie Clohessy1, Prof Theo Arvanitis1, Dr Carla Toro1, Dr Mark Elliott1 carried out this study supported by collaborators Mark Evans2,3 Carly Craddock2,3.

1) Institute of Digital Health care, WMG, University of Warwick 2) The Royal Wolverhampton NHS Trust, Wolverhampton, UK, 3) National Institute for Health Research Clinical Research Network West Midlands, Birmingham, UK).

The purpose of this study is to identify local examples of digital platforms and approaches along with any barriers that may have delayed or stopped use of any digital tools. By taking part in this study, you had the opportunity to provide your contact details. If you chose to do so, we may invite you to take part in a qualitative interview study which will ask you about your experience with digital tools/platforms in further depth. Please be reminded that all data collected will remain confidential and anonymised. As mentioned in the information sheet, please note if you only partially completed the survey, we will still use the information that you provided up to the point that you stopped the survey. The findings of this study will be written up and published in a peer reviewed journal. The data will be stored securely on University of Warwick secure shared servers (M drive) for the standard data retention period of 10 years. The only people able to access the data will be the investigators of the project; Researchers Sophie Clohessy, Professor Theo Arvanitis, Dr Carla Toro, Dr Mark Elliott (Institute Digital Healthcare, WMG, University of Warwick).

For peer review only - http://bmjopen.bmj.com/site/about/guidelines.xhtml

If you have questions or concerns about the study please feel free to contact Sophie Clohessy, email s.clohessy@warwick.ac.uk.

You may wish to keep a copy of this debriefing sheet for your records.

Thank you for taking your time to complete our study, we hope you enjoyed the experience.

Please click on the next page to complete the study.

End of Block: Debriefing sheet

For peer review only

For peer review only - http://bmjopen.bmj.com/site/about/guidelines.xhtml

Supplementary Information 2b - Online Survey: R&D Department Staff

Start of Block: information sheet

Information sheet **PROJECT TITLE**: **Exploring Digital Approaches within R&D Departments**

NAMES OF RESEARCHERS: Sophie Clohessy1, Prof Theo Arvanitis1, Dr Carla Toro1, Dr Mark Elliott1 (1. Institute of Digital Health care, WMG, University of Warwick)

NAMES OF COLLABORATORS: Carly Craddock2,3, Mark Evans2,3 (2. The Royal Wolverhampton NHS Trust, Wolverhampton, UK, 3. National Institute for Health Research Clinical Research Network West Midlands, Birmingham, UK).

This sheet seeks to provide information, and advice, with respect to an individual's participation in support of the specified research project:

1. The study aims to investigate how online and digital tools/methods are being used for clinical, health and social care research. We are particularly interested to determine which approaches work well and which don't and use these findings to share best practice with the clinical, health and social care research community.

2. This study is funded by the National Institute of Health Research Clinical Research Network West Midlands (CRN West Midlands). The funding for this project has been awarded to Sophie Clohessy, Prof Theo Arvanitis, Dr Carla Toro, Dr Mark Elliott (University of Warwick). The project will be led by The University of Warwick.

3. Participation in this research is totally voluntary, and assurances are given to the effect that no negative consequences will arise from refusal to participate in the research project.

4. Your consent for your data to be used in this questionnaire will be gained by your ticking the consent question at the end of this page, so by ticking this box you agree that your submitted data can be used in the aforementioned study.

5. Your data will be collected anonymously, therefore due to the anonymity of the data at source, once completed it is not possible for participants to withdraw their data from the study. However, you will be given the option to provide your name and contact details if you are happy for us to contact you for further information based on your responses. Those who provide these details have the option to withdraw from the study up to 7 days after submission, by contacting

For peer review only - http://bmjopen.bmj.com/site/about/guidelines.xhtml

Sophie Clohessy, s.clohessy@warwick.ac.uk.

6. Each individual is advised to fully consider, with others if necessary and prior to participation, any disadvantages, side effects, risks and/or discomforts that may arise from participation in this research.

7. Unless specifically agreed otherwise, all information will be held as confidential and will not be distributed outside of the research team.

8. Your data may be used as a source for future research, including research work for publication.

This research has been favourably reviewed by the University's Biomedical and Scientific Research Ethics Committee, Approval Reference: BSREC 111/20-21 dated: 17/01/2022. Dissatisfaction with the conduct of this research may be referred to the person below, who is a senior University of Warwick official entirely independent of this study: Head of Research Governance, Research & Impact Services, University House, University of Warwick, Coventry, CV4 8UW; Tel: 024 76 75733; Email: researchgovernance@warwick.ac.uk

---

consentform **Before you continue please understand that you are confirming your agreement with the following:**

○ I give my consent to my data submitted within this questionnaire being used for the purposes stated above including being happy for my data to be used in future research (1)

○ I do not consent, and I do not wish to take part (2)

Skip To: End of Survey If Before you continue please understand that you are confirming your agreement with the following: = I do not consent, and I do not wish to take part

---

Page Break

For peer review only - http://bmjopen.bmj.com/site/about/guidelines.xhtml

End of Block: information sheet

Start of Block: Work in West Midlands

County **Which county in the Clinical Research Network West Midlands is your employing organisation based within?**

▼ Shropshire (1) ... I don't work in any of these counties (7)

Display This Question:

If Which county in the Clinical Research Network West Midlands is your employing organisation based... = I don't work in any of these counties

End of survey **Thank you for your interest in our survey. Unfortunately we are only interested in responses from people who work within the Clinical Research Network West Midlands.**

Please click on the next page to end the survey.

Skip To: End of Survey If Thank you for your interest in our survey. Unfortunately we are only interested in responses from... Is Displayed

End of Block: Work in West Midlands

Start of Block: Current use of digital tools

Use of tools
Please think about the clinical, health or social care research that you are currently working on/have worked on in the last two years. We are interested to learn whether you have used any digital tools in the R & D department you work in. Specifically, we are interested in digital tools used for set up, recruitment of participants and management of clinical research studies.

In this study a digital tool is defined as an alternative to paper-based methods that is IT based or an online platform that aids any aspect of the research study set up, recruitment of participants and management. Electronic screening for participants at site level Remote monitoring via smart phone applications Local social media routes R & D department's website Text alerts
Think about the clinical research studies you have assisted with in your R & D

For peer review only - http://bmjopen.bmj.com/site/about/guidelines.xhtml

department over the past two years. Has your department used digital tools in the set up, recruitment and management/monitoring of clinical research?

○ Yes (1)

○ No (2)

Display This Question:

If Please think about the clinical, health or social care research that you are currently working on... = No

If not used digital

What barriers have you encountered that have prevented you using digital tools in your R & D department? Please select all answer/s that apply.

☐ Concern digital tools would bias participant recruitment (8)

☐ GDPR concerns (10)

☐ Lack of budget (1)

☐ Stick to what worked in the past (2)

☐ Unaware of suitable digital tools (3)

☐ Would require training to use digital tools (7)

☐ Other (9) ____

Display This Question:

If Please think about the clinical, health or social care research that you are currently working on... = No

For peer review only - http://bmjopen.bmj.com/site/about/guidelines.xhtml

Intentions

Please rate the following statement on a 1 - 5 scale (1 "strongly disagree") and (5 "strongly agree")

"I intend to use digital tools for the set up, recruitment and management of future clinical, health or social care research studies"

- ◯ 1 Strongly disagree (1)
- ◯ 2 (2)
- ◯ 3 (3)
- ◯ 4 (4)
- ◯ 5 Strongly agree (5)

Page Break

For peer review only

Display This Question:

If Please think about the clinical, health or social care research that you are currently working on... = No

X→

Opt in non digital **By taking part in this survey, you also have the opportunity to take part in a qualitative one to one interview (most likely conducted remotely and at a time convenient for you). We anticipate the interview will take approximately between 30 and 60 minutes.**

During this interview, we will explore reasons for not using digital tools in set up, recruitment or monitoring of clinical, health or social care research in further depth and your views towards these tools.

If you are interested in taking part, please provide your name and email address below and we will be in touch shortly to provide further details.

- ○ Yes I am interested in providing my contact details (1)
- ○ No, I opt out of taking part in a qualitative interview (2)

Display This Question:

If By taking part in this survey, you also have the opportunity to take part in a qualitative one to... = Yes I am interested in providing my contact details

Full name **Full Name**

____

Display This Question:

If By taking part in this survey, you also have the opportunity to take part in a qualitative one to... = Yes I am interested in providing my contact details

Email address **Email Address**

____

For peer review only - http://bmjopen.bmj.com/site/about/guidelines.xhtml

Display This Question:

If By taking part in this survey, you also have the opportunity to take part in a qualitative one to... = Yes I am interested in providing my contact details

Job title **Job Title**

____

Display This Question:

If By taking part in this survey, you also have the opportunity to take part in a qualitative one to... = Yes I am interested in providing my contact details

Organisation **Which organisation do you work for?**

____

Display This Question:

If By taking part in this survey, you also have the opportunity to take part in a qualitative one to... = No, I opt out of taking part in a qualitative interview

complete survey **Please click on the next page to complete the survey.**

Skip To: End of Survey If Please click on the next page to complete the survey. Is Displayed

End of Block: Current use of digital tools

Start of Block: Introduction

Display This Question:

If Please think about the clinical, health or social care research that you are currently working on... = Yes

Instructions **On the next page we will ask you some questions about you.**

End of Block: Introduction

Start of Block: Demographics

X→

For peer review only - http://bmjopen.bmj.com/site/about/guidelines.xhtml

Job role **What is your job role?**

- ◯ Data Manager (11)
- ◯ Director for Research (6)
- ◯ Lead Research Nurse (9)
- ◯ R&D Manager/Head of R&D (7)
- ◯ Research and Development Facilitator (governance, study set-up) (8)
- ◯ Research Delivery staff (10)
- ◯ Other (please provide further details below) (5)

____

Organisation **What organisation do you work for?**

- ◯ Clinical trial unit (2)
- ◯ Local Authority (6)
- ◯ NHS Trust (4)
- ◯ Primary Care (3)
- ◯ University (1)
- ◯ Other (please provide further details below) (5)

____

Place of work **Please provide the specific place of work (optional)**

____

End of Block: Demographics

For peer review only - http://bmjopen.bmj.com/site/about/guidelines.xhtml

Start of Block: Introduction to blocks

Intro to blocks **We are now going to ask you about digital tools used in R & D. A block of questions will be repeated for three different areas of clinical research studies: Set up Recruitment Management**

End of Block: Introduction to blocks

Start of Block: Set up

Intro set up **We will now ask you questions about digital tools used in R & D for the set up of clinical research studies.**

Page Break

For peer review only

For peer review only - http://bmjopen.bmj.com/site/about/guidelines.xhtml

Set up
Has the Research and Development department you work in used any digital tools to set up studies, for example for feasibility, governance and authorisation (excluding Edge)?

◯ Yes (1)

◯ No (2)

Skip To: End of Block If Has the Research and Development department you work in used any digital tools to set up studies,... = No

Set up examples **If Yes, please list the digital tool/s used by your R & D team to set up studies**

____

____

____

____

____

Page Break

For peer review only - http://bmjopen.bmj.com/site/about/guidelines.xhtml

Intro set up **We will now ask you a series of questions about an example of a digital tool you just provided for set up of clinical research studies.**

Please think about the tool you perceive to be the most effective. Please name the tool below

____

Hear Digital **How did you hear about this tool?**

- ◯ Approached by digital tool provider (1)
- ◯ Developed in house for specific need (2)
- ◯ Recommended by others (3)
- ◯ Saw advertised online/social media (4)
- ◯ Other (5) ____

set up trainng **Did your R & D department require training in order to use this digital tool?**

- ◯ Yes (1)
- ◯ No (2)

Display This Question:

If Did your R & D department require training in order to use this digital tool? = Yes

For peer review only - http://bmjopen.bmj.com/site/about/guidelines.xhtml

training days **If yes, how many days training were required?**

- ◯ < 1 day (1)
- ◯ 1-5 days (2)
- ◯ > 5 days (3)

---

set up novel **Is this digital tool novel/bespoke to your project team or used widely?**

- ◯ Novel/bespoke to my project team (1)
- ◯ Novel/bespoke to my organisation (2)
- ◯ Used widely (3)

Page Break

For peer review only

For peer review only - http://bmjopen.bmj.com/site/about/guidelines.xhtml

Set up advantages **Based on your experience, please list any advantages of this digital tool? (If applicable)**

____

____

____

____

____

Page Break

For peer review only

For peer review only - http://bmjopen.bmj.com/site/about/guidelines.xhtml

set up disadvantages
Based on your experience, please list any disadvantages of this digital tool? (If applicable)

____
____
____
____
____

set up costs **What costs were associated with acquiring this tool?**

- ◯ A one-off up-front fee (e.g. outright purchase) (1)
- ◯ Periodic recurring fee (e.g. monthly/annual license/maintenance fee) (2)
- ◯ No fee (e.g. open source) (3)
- ◯ Other (4) ____
- ◯ I don't know the answer (5)

set up actual costs **Please state any details you have (and can disclose) on actual costs and any overall savings gained or expect to gain**

____
____
____
____
____

For peer review only

For peer review only - http://bmjopen.bmj.com/site/about/guidelines.xhtml

Cost effective **How cost effective would you say this tool is in terms of whether it has added value/efficiency?**

- ◯ Highly Ineffective 1 (1)
- ◯ 2 (2)
- ◯ 3 (3)
- ◯ 4 (4)
- ◯ 5 (5)
- ◯ 6 (6)
- ◯ Highly Effective 7 (7)

---

set up comments **Please provide any further comments or information in the text box below about your digital tool example. Please write n/a in the box if not applicable.**

____

____

____

____

____

End of Block: Set up

Start of Block: Recruitment

Recruitment intro **We will now ask you questions about digital tools used in R & D for participant recruitment.**

For peer review only - http://bmjopen.bmj.com/site/about/guidelines.xhtml

Page Break

For peer review only

For peer review only - http://bmjopen.bmj.com/site/about/guidelines.xhtml

Recruitment
Has the Research and Development department you work in used any digital means to recruit participants (please only include anything that is specific to your organisation and not anything that is mandated/provided by the study team and used study-wide)?

◯ Yes (1)

◯ No (2)

Skip To: End of Block If Has the Research and Development department you work in used any digital means to recruit partici... = No

Recruitment example **If Yes, please list the digital tool/s used by your R & D team to recruit participants?**

____

____

____

____

____

Page Break

For peer review only

For peer review only - http://bmjopen.bmj.com/site/about/guidelines.xhtml

Intro recruitment **We will now ask you a series of questions about an example of a digital tool you just provided for participant recruitment.**

Please think about the tool you perceive to be the most effective. Please name the tool below

____

Recruitment hear **How did you hear about this tool?**

- ◯ Approached by digital tool provider (1)
- ◯ Developed in house for specific need (2)
- ◯ Recommended by others (3)
- ◯ Saw advertised online/social media (4)
- ◯ Other (5) ____

Recruitment training **Did your R & D department require training in order to use this digital tool?**

- ◯ Yes (1)
- ◯ No (2)

Display This Question:

If Did your R & D department require training in order to use this digital tool? = Yes

For peer review only

Recruitment days **If yes, how many days training were required?**

- < 1 day (1)
- 1-5 days (2)
- > 5 days (3)

Recruitment novel **Is this digital tool novel/bespoke to your project team or used widely?**

- Novel/bespoke to my project team (1)
- Novel/bespoke to my organisation (2)
- Used widely (3)

Page Break

For peer review only - http://bmjopen.bmj.com/site/about/guidelines.xhtml

Recruitment advantag **Based on your experience, please list any advantages of this digital tool? (If applicable)**

____

____

____

____

____

Page Break

For peer review only

Recruitment dis
Based on your experience, please list any disadvantages of this digital tool? (If applicable)

____
____
____
____
____

Recruitment costs **What costs were associated with acquiring this tool?**

- ◯ A one-off up-front fee (e.g. outright purchase) (1)
- ◯ Periodic recurring fee (e.g. monthly/annual license/maintenance fee) (2)
- ◯ No fee (e.g. open source) (3)
- ◯ Other (4) ____
- ◯ I don't know the answer (5)

For peer review only

For peer review only - http://bmjopen.bmj.com/site/about/guidelines.xhtml

cost effective **How cost effective would you say this tool is in terms of whether it has added value/efficiency?**

- ◯ Highly Ineffective 1 (1)
- ◯ 2 (2)
- ◯ 3 (3)
- ◯ 4 (4)
- ◯ 5 (5)
- ◯ 6 (6)
- ◯ Highly Effective 7 (7)

Recruitment costs **Please state any details you have (and can disclose) on actual costs and any overall savings gained or expect to gain**

____

____

____

____

____

Recruitment comments **Please provide any further comments or information in the text box below about your digital tool example. Please write n/a in the box if not applicable.**

____

____

____

____

For peer review only - http://bmjopen.bmj.com/site/about/guidelines.xhtml

End of Block: Recruitment

Start of Block: Management

Manage intro **We will now ask you questions about digital tools used in R & D for the management of clinical research studies.**

Page Break

For peer review only

Manage **Has the Research and Development department you work in used any digital means to manage (i.e. progress, automate or alert for study procedures, study monitoring, financial management, study closure etc) a research study/studies (excluding Edge)?**

○ Yes (1)

○ No (2)

Skip To: End of Block If Has the Research and Development department you work in used any digital means to manage (i.e. pr... = No

Manage exmaple **If Yes, please tell us about the digital tool/s used by your R & D team to manage studies**

____

____

____

____

____

Page Break

For peer review only

Manage intro We will now ask you a series of questions about an example of a digital tool you just provided for research study **management**.

Please think about the tool you perceive to be the most effective. Please name the tool below

____

Manage hear **How did you hear about this tool?**

- ◯ Approached by digital tool provider (1)
- ◯ Developed in house for specific need (2)
- ◯ Recommended by others (3)
- ◯ Saw advertised online/social media (4)
- ◯ Other (5) ____

Manage training **Did your R & D department require training in order to use this digital tool?**

- ◯ Yes (1)
- ◯ No (2)

Display This Question:

If Did your R & D department require training in order to use this digital tool? = Yes

For peer review only - http://bmjopen.bmj.com/site/about/guidelines.xhtml

Manage days training **If yes, how many days training were required?**

- ◯ < 1 day (1)
- ◯ 1-5 days (2)
- ◯ > 5 days (3)

Manage novel **Is this digital tool novel/bespoke to your project team or used widely?**

- ◯ Novel/bespoke to my project team (1)
- ◯ Novel/bespoke to my organisation (2)
- ◯ Used widely (3)

Page Break

For peer review only

Manage advantage **Based on your experience, please list any advantages of this digital tool? (If applicable)**

Page Break

For peer review only

Manage disadvantage
Based on your experience, please list any disadvantages of this digital tool? (If applicable)

____

____

____

____

____

Manage costs **What costs were associated with acquiring this tool?**

- ◯ A one-off up-front fee (e.g. outright purchase) (1)
- ◯ Periodic recurring fee (e.g. monthly/annual license/maintenance fee) (2)
- ◯ No fee (e.g. open source) (3)
- ◯ Other (4) ____
- ◯ I don't know the answer (5)

For peer review only

For peer review only - http://bmjopen.bmj.com/site/about/guidelines.xhtml

Cost effective **How cost effective would you say this tool is in terms of whether it has added value/efficiency?**

- ◯ Highly Ineffective 1 (1)
- ◯ 2 (2)
- ◯ 3 (3)
- ◯ 4 (4)
- ◯ 5 (5)
- ◯ 6 (6)
- ◯ Highly Effective 7 (7)

Manage costs **Please state any details you have (and can disclose) on actual costs and any overall savings gained or expect to gain**

____
____
____
____
____

Manage further comme **Please provide any further comments or information in the text box below about your digital tool example. Please write n/a in the box if not applicable.**

____
____
____
____

For peer review only - http://bmjopen.bmj.com/site/about/guidelines.xhtml
For peer review only

End of Block: Management

Start of Block: Opt in interviews

X→

Qualitative survey **By taking part in this survey, you also have the opportunity to take part in a qualitative one to one interview (most likely conducted remotely and at a time convenient for you). We anticipate the interview will take between 30-60 minutes.**

During this interview, we will explore your experiences about using digital tools in clinical research in further depth. We will ask you questions about the information you provided in this survey.

This study is part of a wider project to learn from people who have used digital tools within clinical research (including researchers) in order to learn about the benefits and drawbacks of these methods and inform researchers and related staff about the digital tools available to them.

If you are interested in taking part, please provide your name and email address below and we will be in touch shortly to provide further details.

○ Yes I am interested in providing my contact details and I consent to the answers on this survey being discussed during the interview (1)

○ No, I opt out of taking part in a qualitative interview (2)

Display This Question:

If By taking part in this survey, you also have the opportunity to take part in a qualitative one to... = Yes I am interested in providing my contact details and I consent to the answers on this survey being discussed during the interview

Full name **Full Name**

____

For peer review only - http://bmjopen.bmj.com/site/about/guidelines.xhtml

Display This Question:

If By taking part in this survey, you also have the opportunity to take part in a qualitative one to... = Yes I am interested in providing my contact details and I consent to the answers on this survey being discussed during the interview

Email address **Email Address**

____

Display This Question:

If By taking part in this survey, you also have the opportunity to take part in a qualitative one to... = Yes I am interested in providing my contact details and I consent to the answers on this survey being discussed during the interview

Job role **Job Title**

____

Display This Question:

If By taking part in this survey, you also have the opportunity to take part in a qualitative one to... = Yes I am interested in providing my contact details and I consent to the answers on this survey being discussed during the interview

Name of organisation **Which organisation do you work for?**

____

End of Block: Opt in interviews

Start of Block: Free text box

Further Information **Please use this text box to provide any further information that you think may be relevant about your experience of using digital tools in your role.**

____
____
____
____
____

For peer review only - http://bmjopen.bmj.com/site/about/guidelines.xhtml

comments **Please leave any comments about the study below. In particular, please tell us if there was anything unclear or confusing about the study or any questions that you think we should include next time.**

____

____

____

____

____

End of Block: Free text box

Start of Block: Debriefing sheet

Debriefing sheet
Debriefing sheet

Study title: Digitising Clinical Research

Thank you for taking part in the questionnaire-based study. Researchers Sophie Clohessy1, Prof Theo Arvanitis1, Dr Carla Toro1, Dr Mark Elliott1 carried out this study (1. Institute of Digital Health care, WMG, University of Warwick) with support from collaborators Carly Craddock2,3, Mark Evans2,3 (2. The Royal Wolverhampton NHS Trust, Wolverhampton, UK, 3. National Institute for Health Research Clinical Research Network West Midlands, Birmingham, UK).

The purpose of this study is to identify local examples of digital platforms and approaches along with any barriers that may have delayed or stopped use of any digital tools. By taking part in this study, you had the opportunity to provide your contact details. If you chose to do so, we may invite you to take part in a qualitative interview study which will ask you about your experience with digital tools/platforms in further depth. Please be reminded that all data collected will remain confidential and anonymised. As mentioned in the information sheet, please note if you only partially completed the survey, we will still use the information that you provided up to the point that you stopped the survey. The findings of this study will be written up and published in a peer reviewed journal. The data will be stored securely on University of Warwick secure shared servers (M drive) for the standard data retention period of 10 years. The only people able to access the data will be the investigators of the project; Researchers Sophie Clohessy, Dr Carla Toro, Dr Mark Elliott, Professor Theo Arvanitis (Institute Digital Healthcare, WMG, University of Warwick).

For peer review only

For peer review only - http://bmjopen.bmj.com/site/about/guidelines.xhtml

If you have questions or concerns about the study please feel free to contact Sophie Clohessy, email s.clohessy@warwick.ac.uk.

You may wish to keep a copy of this debriefing sheet for your records.

Thank you for taking your time to complete our study, we hope you enjoyed the experience.

Please click on the next page to complete the study.

End of Block: Debriefing sheet

For peer review only

Supplementary 3a: Research staff– Digitising Research Qualitative Interview Questions

Interview Introduction

Research Fellow (RF) to thank participants for agreeing to participate in this research interview.

RF: Regarding the Participant Information Sheet and Consent Form we sent to you, did you have any questions about the study?

RF will give brief overview of the sections below before interview begins.

Purpose of the study:
To explore your experience of digital tools used in clinical research studies.

Definition:

- A digital tool can be used throughout different stages of clinical research.
- For example, set up, recruitment, participant retention, intervention delivery, data collection, data analysis and perhaps many more.
- Specific examples include; use of electronic health records to screen participants for eligibility for studies. Tools for recruitment of patients might include trial websites, social media, and email campaigns. Tools for data collection might include electronic platforms such as Prolific.

Why are we doing this study?

- This project is conducted by researchers at the Institute of Digital Healthcare, University of Warwick and funded by the NIHR CRN West Midlands.
- The use of digital tools has rapidly increased since the pandemic. Therefore, we are interested in exploring the experiences of stakeholders in the CRN West Midlands when using digital tools in their clinical research.
- We wish to know what has worked well, what not so well when using these tools. We wish to provide other researchers of local examples in the CRN West Midlands as well as recommendations/guidelines for future studies.

Interview Questions for Research staff

RF: I anticipate that the interview will take approximately 45 minutes to one hour. Please remember that we are interested in your experiences with digital tools in clinical research studies and there are no wrong or right answers. Please feel free to ask me to repeat questions if anything is unclear.

RF: We will now start recording the interview. RF to note time interview started recording.

For the purpose of the recording, I can confirm that consent has been taken from the participant and they had a chance to read the information sheet. We will now start the interview questions.

For peer review only

V2
17/08/2022

Section 1 - Opening question

1.1. RF to ask participants if they have consented to providing their name, place of work, and organisation in report? If yes, participant to state those details now.

1.2. In a few sentences, please tell me about your role and a brief description of the main clinical research studies that you have worked on in the past two years (since 2020).

Section 2 – Definition of digital tools

2.1. In the context of clinical research, what does the term 'digital tool' mean to you?
Prompt: How would you describe it to someone with no prior knowledge of the different digital tools used in clinical research?
Prompt: Please provide brief examples *they don't have to be a tool you have personally used.

Section 3- COVID-19 pandemic questions

3.1. Please describe how/if COVID-19 pandemic affected your research?
Prompt- Were the digital tools you provided as examples in the survey implemented due to Covid? Or other reasons? (If used before covid- why were they implemented).
Prompt-Utilise digital tools (or more digital tools) due to COVID instead of paper/non-digital tools?
Prompt- If yes, what were your thoughts on using more digital tools? (e.g., feel ready? Pressured)
Prompt- If yes, please describe the accelerated implementation of digital tools during the pandemic, how did this happen and were there any impacts of this?

Section 4 - Case study of MOST effective tool

In the survey you provided an example of a digital tool which you deemed to be the most effective digital tool you have used.
The example you provided was ………… and end user was …….. (Research Fellow to provide a sentence describing the tool/end user based on their survey answer)
We would now like to ask you some questions about this digital tool and the clinical research study in which it was used.

4.1. Could you provide some background about the clinical research study in which the digital tool was used?
Prompt: Project status? Study objective? Your role in this study?
Prompt: How was the digital tool used in the clinical research study? (Stage of project).

4.2. Please describe the process in which this digital tool was chosen to be used in study? how and why was it chosen?
Prompt: What previous tool/method did it replace? i.e., paper/non digital.
Prompt: Who chose the tool? (i.e., yourself, colleagues). Is there a process for deciding which tool is selected for use?
Prompt: Were other tools considered? If so, why was this tool chosen? E.g., commonly used?
Prompt: What criteria was used to decide whether it was a good tool or not? Was it helpful? How could this be improved?

For peer review only

V2
17/08/2022

Prompt: Do you think there should be a process that researchers could follow when choosing tools.

4.4. You mentioned on the survey that training was required for this digital tool. Please describe what the training entailed? (Participants will only be asked this question if they said they had training for the digital tool example).

Prompt- Training length? What parts were helpful? Could any areas be improved?

4.5. You mentioned on the survey that you received no training for this digital tool (Participants will only be asked this question if they said no to training)

Prompt- If no, why was training not provided? Do you believe it should have been provided? Would this have aided use/performance?

4.6. We are interested in learning about the performance of this digital tool. Please tell me about your experience of using the digital tool?

Research Fellow to immediately mention Prompt- why was this tool selected as most effective? ***Key follow up question** and Prompt- please consider the experience of using the digital tool to other tools (i.e., another digital tool or non-digital/paper-based method). ***Key follow up question.**
Prompt- did you find it easy or difficult to use the tool?
Prompt- what would you consider to be the benefits or drawbacks of this tool? (Particularly in relation to previous tools or non-digital/paper-based tool).
Prompt- did using the tool fulfil the goals of the research? (Please re-state the goals) If no, what alternative tools could have been used?
Prompt: Have you used this tool prior to this project? If yes, how long have you used this tool for?
Prompt- How many people in your team used the tool in the example you provided? (approx.).
Prompt- Can you describe the end user of this tool? Any formal feedback process?

4.7. Please consider if there were any barriers to implementation/usage of this digital tool?

Prompt- Barrier might be technological, logistical, cost, if participant facing-accessibility?
Prompt- If yes, was the barrier overcome? How was this achieved?

4.8. Thinking of this digital tool, please describe any details you have (and can disclose) on actual costs and any overall savings gained or expect to gain?

Prompt- is the tool cost effective?
If participants do not have information on exact costs…do you think that using digital tool makes savings on indirect costs i.e., adds efficiency? Saves time?

4.9. We are interested in your future intentions to use this tool. Thinking of the current tool choice, would you do anything differently? Or utilise the tool again?

For peer review only

V2
17/08/2022

Prompt- Will you continue to use the tool or similar in future projects? Or chose another tool?
Prompt- Why might this be? Please expand

Section 5 - Case study of LEAST effective tool

5.1. In the survey you provided an example of a digital tool which you deemed to be the LEAST effective digital tool you have used.
The example you provided was ……….. and end user was …….. (Research Fellow to provide a sentence describing the tool/end user based on their survey answer)
We would now like to ask you some questions about this digital tool and the clinical research study in which it was used.

5.2. Could you provide some background about the clinical research study in which the digital tool was used?
Prompt: Project status? Study objective? Your role in this study?
Prompt: How was the digital tool used in the clinical research study? (Stage of project).

5.3. Please describe the process in which this digital tool was chosen to be used in the study? how and why was it chosen?
Prompt? Why it was thought to be effective when chosen but turned out not to be
Prompt: What previous tool/method did it replace? i.e., paper/non digital.
Prompt: Who chose the tool? (i.e., yourself, colleagues). Is there a process for deciding which tool is selected for use?
Prompt: Were other tools considered? If so, why was this tool chosen? E.g., commonly used?
Prompt: What criteria was used to decide whether it was a good tool or not? Was it helpful? How could this be improved?
Prompt: Do you think there should be a process that researchers could follow when choosing tools?

5.4. a) You mention on the survey that training was required for this digital tool. Please describe what the training entailed?

Prompt- Training length? What parts were helpful? Could any areas be improved?

b) You mention on the survey that you received no training for this digital tool.

Prompt- If no, why was training not provided? do you believe it should provided? Would this have aided use?/performance?

5.5. We are interested in learning about the performance of this digital tool. Please tell me about your experience of using the digital tool and why it was deemed not effective?
Prompt- why was this tool selected as least effective? ***Key follow up question**
Prompt- please consider the experience of using the digital tool to other tools (i.e., another digital tool or non-digital/paper-based method). ***Key follow up question.**

- Prompt- did you find it easy or difficult to use the tool?

For peer review only

V2
17/08/2022

Prompt- what would you consider to be the benefits or drawbacks of this tool? (Particularly in relation to previous tools or non-digital/paper-based tool).
Prompt- did using the tool fulfil the goals of the research? (Please re-state the goals) If no, what alternative tools could have been used?
Prompt: Have you used this tool prior to this project? If yes, how long have you used this tool for?
How many people in your team used the tool in the example you provided? (approx.).
Experience of end user? Any formal feedback process?

5.6. Please consider if there were any barriers to implementation/usage of this digital tool?
Prompt- Barrier might be technological, logistical, cost, if participant facing, accessibility?
Prompt- How was the barrier overcome? How was this achieved?

5.7. Thinking of this digital tool, please describe any details you have (and can disclose) on actual costs and any overall savings gained or expect to gain?

Prompt- is the tool cost effective?
If participants do not have information on exact costs… do you think that using digital tool makes savings on indirect costs i.e., adds efficiency? Saves time?

5.8. We are interested in your future intentions to use this tool. Thinking of the current tool choice, would you do anything differently? Or utilise the tool again?
Prompt- Are there any changes that could be made to make the tool more effective, if not what other tools could be used an alternative (if any)
Prompt- Will you continue to use the tool or similar in future projects? Or chose another tool?
Prompt- Why might this be? Please expand.

6. General questions about digital tools

6.1. Are there instances in your clinical research studies where digital tools could have been used instead of non-digital/paper-based tools?
Prompt- If yes, why is this? which one? Would implementation of digital tools have affected the outcomes? In what way?

6.2. Please consider if you have encountered any barriers that may have prevented you from using digital tools in your role?
Prompt- Examples might include budget, training, just used what worked before, knowledge.

6.4. When considering different digital tools in clinical research, what do you think would help researchers when deciding which digital tool to use and where might you find this advice?
Prompt- What type of information might be useful? Case studies? Example where things have worked well/not so well? Database of tools? (Including costs, level of difficulty etc).

For peer review only

V2
17/08/2022

Prompt- What evidence of success would you look for when deciding whether to use a particular tool?
Prompt- Where would you expect to find advice? e.g., in house at local CRN or own in house research?

Final thoughts

7.Is there anything I haven't asked you about that you think is important to add? Please tell me…

8. If I need to, would it be possible to come back to you if a further specific question arises as we conduct this research?

9.Thank you so much again for taking part, your time, knowledge and experience is greatly appreciated. Is there anything you would like to ask me about this research?

RF to note time interview ended and stop recording.

For peer review only

Supplementary 3b: Qualitative Interviews - R & D staff

Interview Introduction

Research Fellow (RF) to thank participants for agreeing to participate in this research interview.

RF: Regarding the Participant Information Sheet and Consent sheet we sent to you, did you have any questions about the study?

RF will give brief overview of the sections below before interview begins.

Purpose of the study:
To explore your experience of digital tools used in clinical research studies as an individual working in R & D.

Definition:

- A digital tool can be used throughout different stages of clinical research.
- We are interested in your experiences of using digital tools, specifically, we are interested in digital tools used for set up, recruitment of participants and management of clinical research studies.

Why are we doing this study?

- This project is conducted by researchers at the Institute of Digital Healthcare, University of Warwick and funded by the NIHR CRN West Midlands.
- The use of digital tools has rapidly increased since the pandemic. Therefore, we are interested in exploring the experiences of stakeholders in the CRN West Midlands when using digital tools in their clinical research.
- We wish to know what has worked well, what not so well when using these tools. We wish to provide other researchers and R & D departments local examples in the CRN West Midlands as well as recommendations/guidelines for future studies.

For peer review only

Interview Questions for R & D staff

RF: I anticipate that the interview will take approximately between 45 to 60 minutes. Please remember that we are interested in your experiences with digital tools in clinical research studies and there are no wrong or right answers. Please feel free to ask me to repeat questions if anything is unclear.

RF: We will now start recording the interview. RF to note time interview started recording.

For the purpose of the recording, I can confirm that consent has been taken from the participant and they had a chance to read the information sheet. We will now start the interview questions.

Section 1 - Opening question

1.1. RF to ask participants if they have consented to providing their name, place of work, and organisation in report? If yes, participant to state those details now.

Section 2 – Definition of digital tools

2.1. In the context of clinical research, what does the term 'digital tool' mean to someone working in R & D?
Prompt: How would you describe it to someone with no prior knowledge of the different digital tools used in clinical research?
Prompt: Please provide brief examples *they don't have to be tool you have personally used. Can you provide any examples of the tools someone working in an R & D department might encounter?

Section 3- COVID-19 pandemic questions

In the survey you provided an example of an effective tool used in set up, recruitment, management of clinical research.

3.1. Please describe how/if COVID-19 pandemic affected your research, specifically with reference to your use of digital tools?
Prompt- Were the digital tools you mentioned in the survey implemented due to Covid? Or other reasons? (If used before covid- why were they implemented).
Prompt-Utilise digital tools (or more digital tools) due to COVID instead of paper/non-digital tools?
Prompt- If yes, what were your thoughts on using more digital tools? (e.g., feel ready? Pressured)
Prompt- If yes, please describe the accelerated implementation of digital tools during the pandemic, how did this happen and were there any impacts of this?

Section 4 - Case study most effective tool used for set up

In the survey you provided an example of a digital tool which you deemed to be the most effective digital tool you have used for the set-up of clinical research studies.
The example you provided was ………… (Research Fellow to provide a sentence describing the tool based on their survey answer)

For peer review only

We would now like to ask you some questions about this digital tool and the clinical research/trial in which it was used.

4.1. Could you provide some background about how the digital tool was used?
Prompt: Briefly describe the tool and how was it used in a study/trial? Your role/how much did you interact with tool?

4.2. Please describe the process in which this digital tool was chosen? how and why was it chosen?
Prompt: What previous tool/method did it replace? i.e., paper/non digital.
Prompt: Who chose the tool? (i.e., yourself, colleagues). Is there a process for deciding which tool is selected for use?
Prompt: Were other tools considered? If so, why was this tool chosen? E.g., commonly used?
Prompt: What criteria was used to decide whether it was a good tool or not? Was it helpful? How could this be improved?
Prompt: Do you think there should be a process that researchers could follow when choosing tools.

4.3. You mentioned on the survey that training was required for this digital tool. Please describe what the training entailed?
Prompt- Training length? What parts were helpful? Could any areas be improved?

4.4. You mentioned on the survey that you received no training for this digital tool.
Prompt- If no, why was training not provided? Do you believe it should have been provided? Would this have aided use/performance?

4.5. We are interested in learning about the performance of this digital tool. Please tell me about your experience of using the digital tool?

Prompt- Why was this tool selected as most effective? ***Key follow up question**
Prompt- Please consider the experience of using the digital tool to other tools (i.e., another digital tool or non-digital/paper-based method). ***Key follow up question.**
Prompt- Did you find it easy or difficult to use the tool?
Prompt- What would you consider to be the benefits or drawbacks of this tool? (Particularly in relation to previous tools or non-digital/paper-based tool).
Prompt- Did using the tool fulfil the goals of the research? (Please re-state the goals) If no, what alternative tools could have been used?
Prompt: Have you used this tool prior to this project? If yes, how long have you used this tool for?
Prompt-How many people in your team used the tool in the example you provided? (approx.).
Can you describe the end user of this tool? Any formal feedback process?

4.6. Please consider if there were any barriers to implementation/usage of this digital tool?
Prompt- Barrier might be technological, logistical, cost, if participant facing-accessibility?
Prompt- If yes, was the barrier overcome? How was this achieved?

For peer review only

4.7. Thinking of this digital tool, please describe any details you have (and can disclose) on actual costs and any overall savings gained or expect to gain?
Prompt- Is the tool cost effective?
Prompt- If participants do not have information on exact costs… do you think that using digital tool makes savings on indirect costs i.e., adds efficiency? saves time?

4.8. We are interested in your future intentions to use this tool. Thinking of the current tool choice, would you do anything differently? Or utilise the tool again?
Prompt- Will you continue to use the tool or similar in future projects? Or chose another tool?
Prompt- Why might this be? Please expand.

Section 5- Case study most effective tool used for recruitment

Section 6- Case study most effective tool used for management

7. General questions about digital tools

7.1. Are there instances in your clinical research studies where digital tools could have been used instead of non-digital/paper-based tools?
Prompt- If yes, why is this? which one? Would implementation of digital tools have affected the outcomes? In what way?

7.2. Please consider if you have encountered any barriers that may have prevented you from using digital tools in your role?
Prompt- Examples might include budget, training, just used what worked before, knowledge.

7.3. When considering different digital tools in clinical research, what do you think would help researchers when deciding which digital tool to use and where might you find this advice?
Prompt- What type of information might be useful? Case studies? Example where things have worked well/not so well? Database of tools? (Including costs, level of difficulty etc).
Prompt- What evidence of success would you look for when deciding whether to use a particular tool?
Prompt- Where would you expect to find advice? e.g., in house at local CRN or own in house research?

Final thoughts

Is there anything I haven't asked you about that you think is important? Please tell me…

If I need to, would it be possible to come back to you if a further specific question arises as we conduct this research?

For peer review only

Thank you so much again for taking part, your time, knowledge and experience is greatly appreciated. Is there anything you would like to ask me about this research?

RF to note time interview ended and stop recording.

For peer review only

Standards for Reporting Qualitative Research (SRQR)*

http://www.equator-network.org/reporting-guidelines/srqr/

Title and abstract

	Page/line no(s).
**Title** - Concise description of the nature and topic of the study Identifying the study as qualitative or indicating the approach (e.g., ethnography, grounded theory) or data collection methods (e.g., interview, focus group) is recommended	1
**Abstract** - Summary of key elements of the study using the abstract format of the intended publication; typically includes background, purpose, methods, results, and conclusions	1-2

Introduction

**Problem formulation** - Description and significance of the problem/phenomenon studied; review of relevant theory and empirical work; problem statement	Pg3, L9-19
**Purpose or research question** - Purpose of the study and specific objectives or questions	Pg4 L1-4

Methods

**Qualitative approach and research paradigm** - Qualitative approach (e.g., ethnography, grounded theory, case study, phenomenology, narrative research) and guiding theory if appropriate; identifying the research paradigm (e.g., postpositivist, constructivist/ interpretivist) is also recommended; rationale**	Pg7 L15-16
**Researcher characteristics and reflexivity** - Researchers' characteristics that may influence the research, including personal attributes, qualifications/experience, relationship with participants, assumptions, and/or presuppositions; potential or actual interaction between researchers' characteristics and the research questions, approach, methods, results, and/or transferability	Not Included
**Context** - Setting/site and salient contextual factors; rationale**	P7 L16-20
**Sampling strategy** - How and why research participants, documents, or events were selected; criteria for deciding when no further sampling was necessary (e.g., sampling saturation); rationale**	P6 L24-25
**Ethical issues pertaining to human subjects** - Documentation of approval by an appropriate ethics review board and participant consent, or explanation for lack thereof; other confidentiality and data security issues	P26, L29-31
**Data collection methods** - Types of data collected; details of data collection procedures including (as appropriate) start and stop dates of data collection and analysis, iterative process, triangulation of sources/methods, and modification of procedures in response to evolving study findings; rationale**	P7 L1-20

For peer review only - http://bmjopen.bmj.com/site/about/guidelines.xhtml

**Data collection instruments and technologies** - Description of instruments (e.g., interview guides, questionnaires) and devices (e.g., audio recorders) used for data collection; if/how the instrument(s) changed over the course of the study	P7 L4-10
**Units of study** - Number and relevant characteristics of participants, documents, or events included in the study; level of participation (could be reported in results)	P4 Table 1
**Data processing** - Methods for processing data prior to and during analysis, including transcription, data entry, data management and security, verification of data integrity, data coding, and anonymization/de-identification of excerpts	P15 L3-9
**Data analysis** - Process by which inferences, themes, etc., were identified and developed, including the researchers involved in data analysis; usually references a specific paradigm or approach; rationale**	P7 L15-16
**Techniques to enhance trustworthiness** - Techniques to enhance trustworthiness and credibility of data analysis (e.g., member checking, audit trail, triangulation); rationale**	P7 L16-19

Results/findings

**Synthesis and interpretation** - Main findings (e.g., interpretations, inferences, and themes); might include development of a theory or model, or integration with prior research or theory	P15 – P22
**Links to empirical data** - Evidence (e.g., quotes, field notes, text excerpts, photographs) to substantiate analytic findings	P15 Table 5 + Supplementary Information

Discussion

**Integration with prior work, implications, transferability, and contribution(s) to the field -** Short summary of main findings; explanation of how findings and conclusions connect to, support, elaborate on, or challenge conclusions of earlier scholarship; discussion of scope of application/generalizability; identification of unique contribution(s) to scholarship in a discipline or field	P22-24
**Limitations** - Trustworthiness and limitations of findings	P24, L14-25

Other

**Conflicts of interest** - Potential sources of influence or perceived influence on study conduct and conclusions; how these were managed	P26 L21-25
**Funding** - Sources of funding and other support; role of funders in data collection, interpretation, and reporting	P26 L17-19

*The authors created the SRQR by searching the literature to identify guidelines, reporting standards, and critical appraisal criteria for qualitative research; reviewing the reference lists of retrieved sources; and contacting experts to gain feedback. The SRQR aims to improve the transparency of all aspects of qualitative research by providing clear standards for reporting qualitative research.

For peer review only - http://bmjopen.bmj.com/site/about/guidelines.xhtml

**The rationale should briefly discuss the justification for choosing that theory, approach, method, or technique rather than other options available, the assumptions and limitations implicit in those choices, and how those choices influence study conclusions and transferability. As appropriate, the rationale for several items might be discussed together.

Reference:
O'Brien BC, Harris IB, Beckman TJ, Reed DA, Cook DA. **Standards for reporting qualitative research: a synthesis of recommendations.** *Academic Medicine*, Vol. 89, No. 9 / Sept 2014 DOI: 10.1097/ACM.0000000000000388

For peer review only

For peer review only - http://bmjopen.bmj.com/site/about/guidelines.xhtml
